# Calorie restriction modulates the transcription of genes related to stress response and longevity in human muscle: The CALERIE study

**DOI:** 10.1111/acel.13963

**Published:** 2023-10-12

**Authors:** Jayanta Kumar Das, Nirad Banskota, Julián Candia, Michael E. Griswold, Melissa Orenduff, Rafael de Cabo, David L. Corcoran, Sai Krupa Das, Supriyo De, Kim Marie Huffman, Virginia B. Kraus, William E. Kraus, Corby K. Martin, Susan B. Racette, Leanne M. Redman, Birgit Schilling, Daniel W. Belsky, Luigi Ferrucci

**Affiliations:** ^1^ Longitudinal Studies Section, Translation Gerontology Branch National Institute on Aging, National Institutes of Health Baltimore Maryland USA; ^2^ Computational Biology and Genomics Core National Institute on Aging, National Institutes of Health Baltimore Maryland USA; ^3^ MIND Center, UMMC School of Medicine Jackson Mississippi USA; ^4^ Duke Molecular Physiology Institute and Department of Medicine Duke University School of Medicine Durham North Carolina USA; ^5^ Translation Gerontology Branch, National Institute on Aging National Institutes of Health Baltimore Maryland USA; ^6^ Department of Genetics University of North Carolina at Chapel Hill Chapel Hill North Carolina USA; ^7^ Energy Metabolism, Jean Mayer USDA Human Nutrition Research Center on Aging Tufts University Boston Massachusetts USA; ^8^ Pennington Biomedical Research Center Louisiana State University Baton Rouge Louisiana USA; ^9^ College of Health Solutions Arizona State University Phoenix Arizona USA; ^10^ The Buck Institute for Research on Aging Novato California USA; ^11^ Department of Epidemiology & Butler Columbia Aging Center Columbia University Mailman School of Public Health New York City New York USA

**Keywords:** calorie restriction, FOXO, heat shock response, inflammation, mitochondrial biogenesis, skeletal muscle, splicing

## Abstract

The lifespan extension induced by 40% caloric restriction (CR) in rodents is accompanied by postponement of disease, preservation of function, and increased stress resistance. Whether CR elicits the same physiological and molecular responses in humans remains mostly unexplored. In the CALERIE study, 12% CR for 2 years in healthy humans induced minor losses of muscle mass (leg lean mass) without changes of muscle strength, but mechanisms for muscle quality preservation remained unclear. We performed *high*‐depth RNA‐Seq (387–618 million paired reads) on human vastus lateralis muscle biopsies collected from the CALERIE participants at baseline, 12‐ and 24‐month follow‐up from the 90 CALERIE participants randomized to CR and “ad libitum” control. Using linear mixed effect model, we identified protein‐coding genes and splicing variants whose expression was significantly changed in the CR group compared to controls, including genes related to proteostasis, circadian rhythm regulation, DNA repair, mitochondrial biogenesis, mRNA processing/splicing, FOXO3 metabolism, apoptosis, and inflammation. Changes in some of these biological pathways mediated part of the positive effect of CR on muscle quality. Differentially expressed splicing variants were associated with change in pathways shown to be affected by CR in model organisms. Two years of sustained CR in humans positively affected skeletal muscle quality, and impacted gene expression and splicing profiles of biological pathways affected by CR in model organisms, suggesting that attainable levels of CR in a lifestyle intervention can benefit muscle health in humans.

## Graphical abstract

Two years of sustained CR in humans positively affected skeletal muscle quality, and impacted gene expression and splicing profiles of biological pathways known to affected by CR in model organisms. CR involved genes related in proteostasis, circadian rhythm regulation, DNA repair, mitochondrial biogenesis, mRNA processing/splicing, FOXO3 metabolism, apoptosis, and inflammation. Alternative splicing plays a key role in regulating key biological mechanisms targeted by CR.

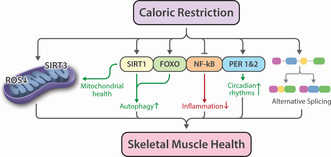

**Abbreviations**
AL: ad libitum
BMI: body mass index
CALERIE: comprehensive assessment of long‐term effects of reducing intake of energy
CR: calorie restriction
DGE: differential gene expression
DXA: dual‐energy x‐ray absorptiometry

## 1 INTRODUCTION

Across animal species, aging is associated with typical anatomical and physiological changes in multiple tissues and organs, as well as with pathology and functional impairments; these eventually lead to disability and death (Blagosklonny & Hall, 2009). Calorie restriction (CR), the most‐studied non‐genetic intervention to counter the effects of aging, increases health span and longevity in most model organisms from yeast to rodents (López‐Lluch & Navas, 2016; Most et al., 2017). The health benefits of CR are conserved in non‐human primates; whether CR extends health and life span in humans is uncertain (Anderson et al., 2018; Mattison et al., 2017). The Comprehensive Assessment of Long term Effects of Reducing Intake of Energy (CALERIE™) was a randomized controlled trial sought to assess the effects of reducing calorie intake by 25% for 2 years while maintaining a normal intake of essential nutrients, in young and middle‐aged nonobese individuals compared to an “ad libitum” (AL) control group (Bales & Kraus, 2013; Di Francesco et al., 2018). The CALERIE intervention resulted in ~12% CR, which was accompanied by 10.4% sustained weight loss over the 2 years. Analysis of a subset of participants who underwent repeated whole‐body MRI indicated preferential loss of adipose tissue, less visceral fat accumulation and minor but significant loss of muscle tissue (Shen et al., 2021). In addition, CR compared to AL improved cardiometabolic risk profile and reduced blood pressure without any adverse effects on the quality of life (Kraus et al., 2019; Martin et al., 2016). The decline of muscle mass was not associated with significant decline of muscle strength, suggesting that CR improved muscle health. Whether biological mechanisms activated by CR and underlying such improvements are similar to those observed in animal models remain unknown (Racette et al., 2017).

In previous studies in model organisms, CR positively affects skeletal muscle by improving satellite cell proliferative capacity (Cerletti et al., 2012), reducing mitochondrial proton leak and reactive oxygen species production (Bevilacqua et al., 2004), preventing muscle fiber loss (McKiernan et al., 2004), and improving mitochondrial electron transport chain efficiency (Choi et al., 2011). Some of the effects of CR appear to be mediated by pre‐mRNA processing and nonsense‐mediated decay of mRNA related to aging (Sparks et al., 2017; Tabrez et al., 2017). For example, in rhesus monkeys, CR significantly affects hepatic RNA processing mechanisms (Rhoads et al., 2018); in *C. elegans*, the effect of CR on TORC1 is mediated by increased transcription of splicing factor 1, with the effect of CR on longevity eliminated by inhibiting this mechanism (Heintz et al., 2017). Finally, dietary restriction in ILSXISS mice is associated with widespread changes in splicing regulatory factor expression levels (Lee et al., 2019). Strong evidence that CR affects the expression of circadian clock genes in mammals and flies has led to the hypothesis that circadian clocks might be involved in the beneficial effect of CR (Acosta‐Rodríguez et al., 2022; Astafev et al., 2017; Katewa et al., 2016; Patel et al., 2016).

To date, the sparse information available in humans has been gathered in cross‐sectional studies or small, short‐term randomized studies. A comparison of muscle biopsies from individuals who had been following a CR diet voluntarily for several years versus age‐matched individuals on a Western diet showed that CR enhances proteostasis and cellular quality‐control processes (Yang et al., 2016). A six‐month CR intervention increases mitochondrial biogenesis and reduced DNA damage in young, nonobese adults (Civitarese et al., 2007). CR also has been shown to increase increases mitochondrial content and electron transport chain efficiency and fatty acid oxidation enzyme activities in overweight and obese older individuals (Menshikova et al., 2018; Sparks et al., 2017).

To shed light on mechanisms by which CR may improve muscle health, we performed a comprehensive high‐depth RNA sequencing (RNA‐Seq) analysis of skeletal muscle collected in a selected group for eligible study participants in CALERIE 2. Our aim was to explore gene expression and splicing variants changes induced by CR compared to AL group over 12 and 24‐month. We also aimed to investigate the impact of biological pathways and their mediation effect on CR on muscle quality, operationalized as mass‐adjusted muscle strength.

## 2 RESULTS

### 2.1 Characteristics of CALERIE muscle biopsy study participants

**FIGURE 1:**
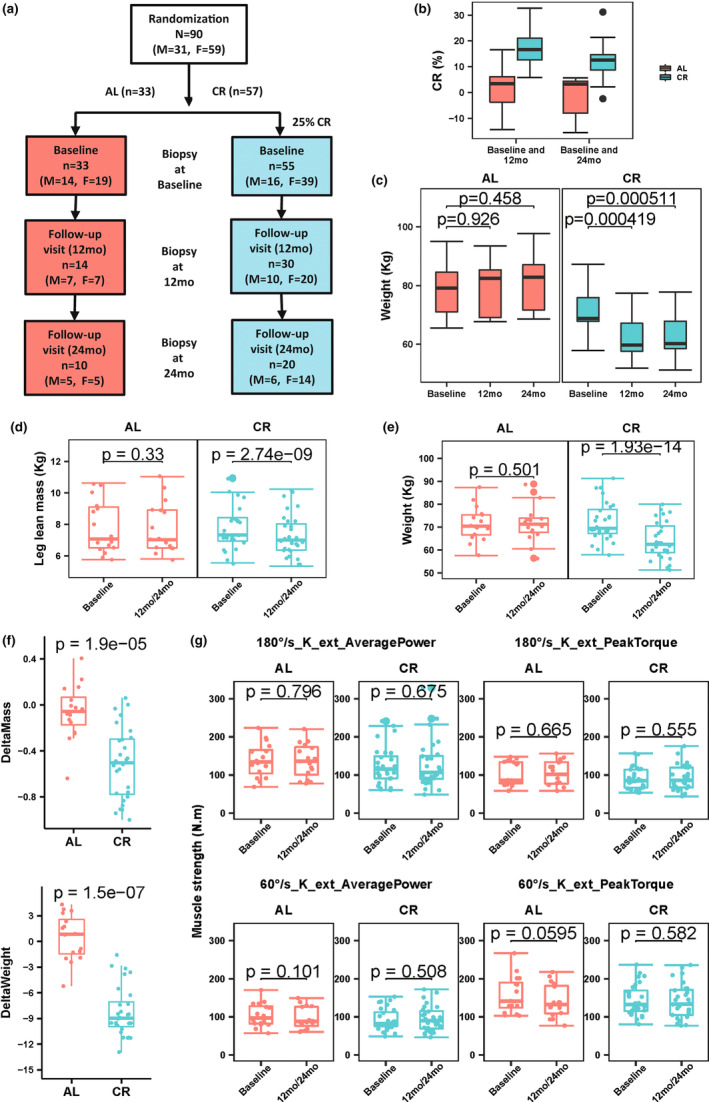
Flowchart for CALERIE participants who underwent muscle biopsies and percentage change of calorie intake and change of muscle mass, weight, and muscle strength in CR and AL. (a) The flow‐chart shows the 90 CALERIE participants randomly assigned to a CR and AL who underwent at least one muscle biopsy (baseline, 12‐month [12 mo], 24‐month [24 mo]). Of note, two individuals in the CR group underwent a muscle biopsy starting at the 12‐month follow‐up only. (b) Box plot showing percentage calorie intake reduction between baseline and 12‐month (*n* = 24 for CR group, *n* = 13 for AL group) as well as baseline and 24‐month (*n* = 17 for CR group, *n* = 7 for AL group) in CALERIE participants. (c) Boxplot showing weight at baseline (Ba), 12‐month follow‐up, and 24‐month follow‐up. Data presented are for participants who participated in muscle biopsy program at baseline and both follow‐up visit (*n* = 19 for CR group and *n* = 7 for AL group). Change of muscle mass (leg lean mass) (d) and weight (e) between baseline and 12‐month/24‐month for participants with muscle biopsy samples at baseline and at least one follow‐up visits at 12‐ or 24‐month (*n* = 27 for CR group and *n* = 15 for AL group). Analysis showed significant changes of muscle mass and weight in the CR group only (paired *t*‐test). (f) Changes of muscle mass and weight were significantly different between CR ad AL (unpaired, Wilcoxon test). (g) Comparison of muscle strength variables for knee extension (absolute average power and peak torque) in isokinetic dynamometry at 60 and 180°s^−1^ between baseline and 12‐month/24‐month (paired *t*‐test). There was no significant change in strength either in the CR and AL group.

A total of 90 individuals (31 men and 59 women) in two groups (*n* = 57 for CR, *n* = 33 for AL) consented to muscle biopsy, for a total of 162 muscle biopsies over the 2‐year study period (Figure 1a). Participants enrolled were healthy with mean (±SD) age 38.4 (±7.3) years for CR and 40.1 (±6.5) years for AL (Table 1). There was no significant difference in mean age, BMI, or weight between completers (participated at baseline and both follow‐up visits) and non‐completers (participated at baseline but refused the muscle biopsy at 12‐ and/or 24‐month) in either AL or CR (Table S1). Average times between biopsies for both CR and AL completer group were 1.1 years for the 12‐month follow‐up and 2.1 years for the 24‐month follow‐up, respectively. The subset of CR participants included in this study achieved ~15% calorie reduction on average between baseline and 12‐month (*n* = 24) and ~12% calorie reduction between baseline and 24‐month (*n* = 17), similar to what was described for the whole CALERIE population (Shen et al., 2021; Figure 1b). Changes in weight were computed for participants who underwent muscle biopsy at both follow‐up visits (*n* = 19 for CR group, *n* = 7 for AL group). CR participants experienced significant (*p* < 0.001) weight loss at 12 months, but there was no further weight loss over the subsequent 12 months, whereas AL participants maintained a stable weight (Figure 1c).

**TABLE 1:** Quantitative characteristics of CALERIE muscle study participants at baseline.

Characteristics	CR group	AL group
Participant (*N*):	57^a^	33
Age (years): Mean (±SD)	38.4 (±7.3)	40.1 (±6.5)
BMI (kg/m^2^): Mean (±SD)	25.4 (±1.7)	25.3 (±1.8)
Weight (kg): Mean (±SD)	72.6 (±8.4)	72.9 (±10.2)
BMI class: %		
Normal weight (lean)	45	45
Sex: %		
Male	29.09	42.42
Female	70.91	57.58
Race: %		
White	76.36	81.82
Asian	10.91	0.00
Black	7.27	12.12
Other	5.45	6.06

^a^ Two participants from the CR group did not consent to muscle biopsy at baseline.

Changes of muscle mass (leg lean mass by DXA), weight, and two muscle strength variables (average power and peak torque in isokinetic dynamometry at 60 and 180°s^−1^) for knee extension were analyzed for the subset of participants (*n* = 28 for CR group and *n* = 16 for AL group) who underwent muscle biopsy at baseline and at least one follow‐up visit (see Section 4). Similar to results reported for the whole CALERIE cohort (Racette et al., 2017), participants in the CR group who consented to muscle biopsy also experienced a decline in leg muscle mass and body weight (Figure 1d–f). For the whole CALERIE cohort, absolute muscle strength (peak torque) declined in the CR group (Racette et al., 2017), whereas in the current subset of participants with muscle biopsy samples, we did not observe significant change in muscle strength (absolute peak torque and average power) in either the CR or AL group (Figure 1g). However, after adjusting for change in muscle mass (leg lean mass), CR was associated with a lower decline compared to AL in the isokinetic task both for average power (*β* = 1.1033, *p* = 0.0058 at 60°s^−1^ and *β* = 0.7239, *p* = 0.0702 at 180°s^−1^) and peak torque (*β* = 0.9865, *p* = 0.0144 at 60°s^−1^ and *β* = 0.7844, *p* = 0.0430 at 180°s^−1^).

### 2.2 Differential changes of gene expression analysis linear over time

**FIGURE 2:**
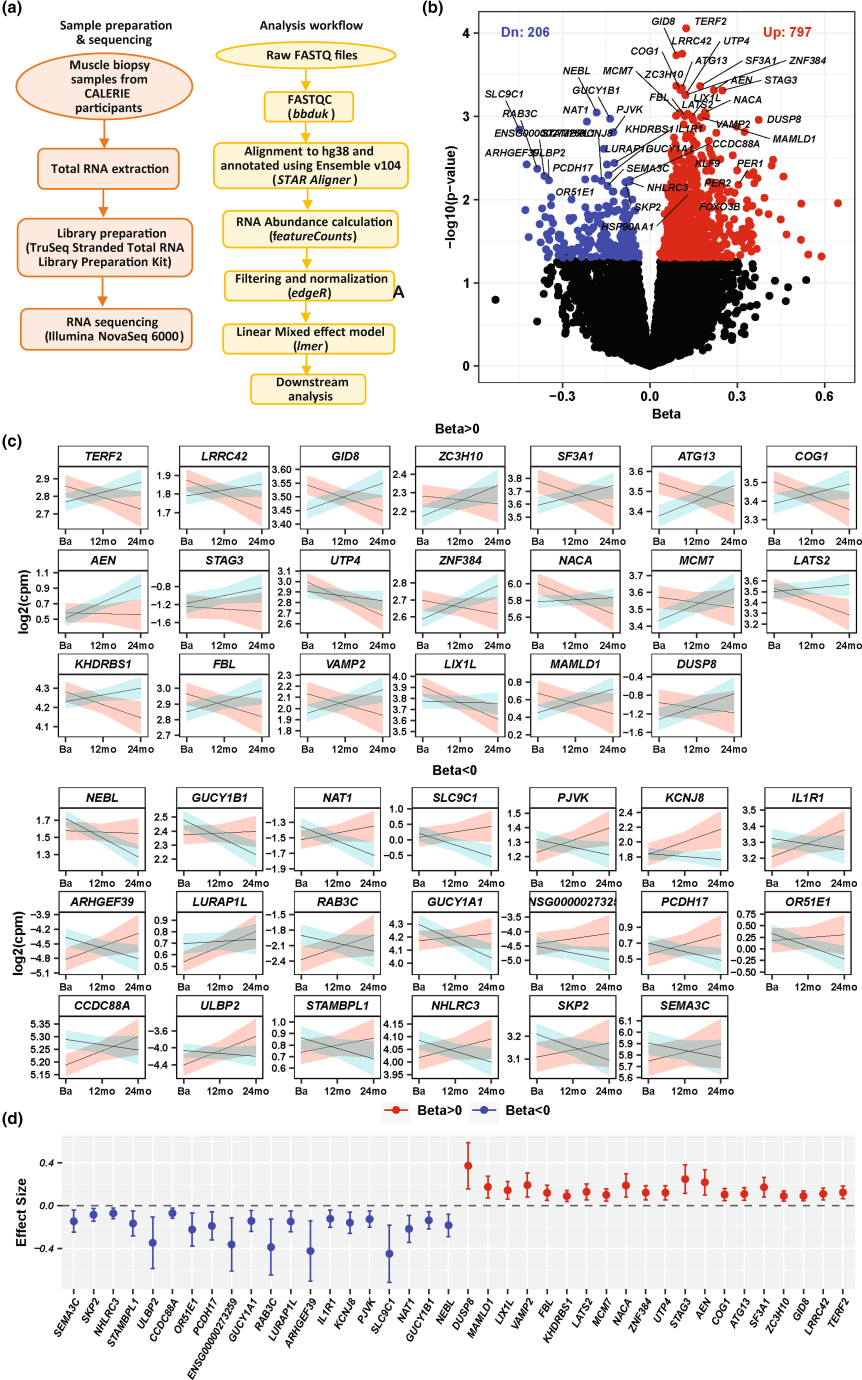
Overview of analysis workflow and quantification of differentially expressed protein‐coding RNAs produced by linear mixed‐effects modeling (LMM) with linear time dependency. (a) A flow‐chart diagram depicting sample preparation and analysis workflow. (b) A Volcano plots depicting results of differential changes of gene expression between CR and AL using LMM and linear time. Red dot (positive beta: *β*+) represents genes whose differential expression is significantly increase in CR compared to AL over time; blue dot (for negative beta: *β*−) represents genes whose expression is significantly decreased in CR compared to AL over time. Some of the genes in each group (*β*+ and *β*−) were labeled. (c) Linear smooth trajectory (unadjusted) drawn with 95% confidence interval for the 20 top differentially expressed genes that became significantly overexpressed (left) and underexpressed genes (right) with time in CR compared to AL; in differentially trajectories, light blue colors are for CR and light red color are for AL; *x*‐axis represents three timepoints: baseline (Ba), 12‐month (12 mo), and 24‐month(24 mo), and *y*‐axis represents RNA expression (log2(CPM)). (d) Forest plot of top 40 transcripts showing range of beta coefficient (*β*) (20 with *β*+ and 20 with *β*−) and standard errors.

A workflow diagram of the study is shown in Figure 2a. Across 162 samples from 90 individuals, we obtained RNAs from 60,605 genes. RNAs for non‐protein‐coding genes (*n* = 40,672) and those with read counts of 0 in ≥50% of total samples were excluded (~17.5%), leaving 16,450 RNAs for protein coding genes for the analysis corresponding to 82.5% of known protein‐coding genes of 19,937 with human genome 38 (hg38) with Ensembl v104. We analyzed differential change in gene expression between CR and AL groups by linear mixed effect models (see Section 4). These models assume a linear change in gene expression in response to CR during the entire 24‐month CALERIE™ intervention. We identified 1003 differentially expressed genes between CR and AL at the threshold of *p* < 0.05; 797 genes were overexpressed (positive beta) and 206 genes underexpressed (negative beta) in CR compared to AL (Figure 2b). The trajectories of change in the top 20 over and underexpressed genes are shown in Figure 2d. We excluded possible violations of the linear change assumption in analyses that separately considered changes that occurred over the first 12 months and over the subsequent 12 months (24 months); results from 24 months were very consistent and captured with those obtained under the linear assumption over 2‐year follow‐up visit (Figure S1).

For in‐depth characterization, we focused the differential gene expression (DGE) analysis on the top set of the 199 genes (out of 1003 as mentioned above) whose expression changed differentially over time at the threshold of *p* < 0.01 (Table S2). Of these, 171 were overexpressed and 28 underexpressed in CR compared to AL (Table S3, Figure S2); the majority of these genes were previously identified as affected by CR in model organisms and point to stress response mechanisms that are considered hallmarks of aging (Table 2). In Table 2, the list includes genes that code for proteins of the following processes: the sheltering complex that protect telomeres (*TERF2*, *TINF2*); molecular chaperones (*HSP90AA1*, *HSPA5*); autophagy (*ATG13*, *TBC1D14*) and integrated stress response (*TAF4*, *GCN1*); mRNA processing and splicing factors (*SF3A1*, *KHDRBS1*, *SF3B4*, *PRF8*, *WBP11*, *PATL1*, *PRPF6*, *SNRNP200*, *AAR2*, *ELAVL1*); circadian clocks (*KLF15*, *KLF9*, *USP2*, *PER1*, *PER2*, *SUV39H1*, *SIK3*, *CRY2*); mitochondrial integrity and function (*ZC3H10*, *RNF185*, *PPRC1*, *PDP2*, *PLD6*); DNA damage repair (*SSRP1*, *USP11*); promoters of genomic stability (*MCM7*, *PKNOX1*); muscle growth and repair (*NACA*, *CERS2*) and downregulated in CR, inflammation (*IL1‐R1*, *LURAP1*). We also performed a correlation study between gene expression and muscle strength values at baseline. As reported in Table 2, a group of genes showed relatively low correlation with the muscle strength at baseline, although some of these correlations were statistically significant (Figure S3).

**TABLE 2:** Genes showing differential expression changes in CR compared to AL participants during the 24‐month CR intervention.

Symbol	Name	Characteristics and functions	References
(A) Genes with increased expression under CR (*p* < 0.01)			
*TERF2*	Telomeric repeat binding factor 2	A component of the telomere nucleoprotein sheltering complex that plays a key role in the protective activity of telomeres	Smogorzewska et al. (2000)
*ZC3H10*	Zinc finger CCCH‐type containing 10	DNA‐binding transcription factor that activates UCP1 increases thermogenic gene expressions in BAT and iWAT in vivo	Nguyen et al. (2020) and Yi et al. (2019)
*SF3A1*	Splicing factor 3a subunit 1	Spliceosome protein	Rhoads et al. (2018)
*ATG13*	Autophagy related 13	An autophagy factor and a target of the TOR kinase signaling pathway. Essential for autophagosome formation and mitophagy	Jung et al. (2009) and Ntsapi and Loos (2016)
*NACA*	Nascent polypeptide associated complex subunit alpha	Regulates postnatal skeletal muscle growth and regeneration	Park et al. (2010)
*MCM7*	Minichromosome maintenance complex component 7	Declines with aging in human fibroblasts, prevents genomic instability during replication and its expression is reduced in replicative senescence	Tsitsipatis et al. (2022)
*KHDRBS1*	KH RNA‐binding domain containing, signal transduction associated 1	RNA‐binding protein family with many functions including alternative splicing and cell cycle regulation. Upregulated by CR in muscle of adult cynomolgus monkeys (*Macaca fascicularis*)	Wang et al. (2009)
*SF3B4*	Splicing factor 3b subunit 4	Involved in pre‐mRNA splicing as a component of the splicing factor SF3B complex	Qin et al. (2016)
*PKNOX1*	PBX/knotted 1 homeobox 1	Improves genomic stability “in vitro”	Iotti et al. (2011)
*PDP2*	Pyruvate dehydrogenase phosphatase catalytic subunit 2	Mitochondrial protein that enhances the utilization of pyruvate in oxidative phosphorylation	Wiley and Campisi (2016)
*PRPF8*	Pre‐mRNA processing factor 8	Core component of U2‐type and the minor U12‐type spliceosomes	Le Couteur et al. (2021), Rhoads et al. (2018), and Zhang et al. (2019)
*WBP11*	WW domain‐binding protein 11	Colocalizes with mRNA splicing factors and intermediate filament‐containing perinuclear networks	Ubaida‐Mohien et al. (2019)
*RNF185*	Ring finger protein 185	E3 ubiquitin‐protein ligase that regulates selective mitochondrial autophagy. Protects cells from ER stress‐induced apoptosis by increasing the degradation of misfolded proteins that accumulate in the endoplasmic reticulum (ER) for ubiquitination and subsequent proteasome‐mediated degradation	Tang et al. (2011)
*TAF4*	TATA‐box binding protein associated factor 4	Inhibits polysome formation in condition of stress	Molenaars et al. (2018) and Wright et al. (2006)
*PATL1*	PAT1 homolog 1, processing body mRNA decay factor	Coordinate the assembly and activation of a decapping messenger ribonucleoprotein (mRNP) that promotes 5′‐3′ mRNA degradation	Vindry et al. (2017)
*PPRC1*	PPARG related coactivator 1	Similar to PPAR‐gamma coactivator 1 (PGC‐1α) activates mitochondrial biogenesis by interacting with nuclear respiratory factor 1 (NRF1)	Miller et al. (2019) and Shi et al. (2021)
*PRPF6*	Pre‐mRNA processing factor 6	A key spliceosome proteins that is upregulated with CR	Le Couteur et al. (2021)
*PIAS3*	Protein inhibitor of activated STAT 3	Protects STAT3 from overactivation, and considered a “fine tuning tool” for the modulation of NFκB, SMAD, and MITF	Yagil et al. (2010)
*PLD6*	Phospholipase D family member 6	Located in mitochondrial outer membrane enables cardiolipin hydrolase activity and regulates mitochondrial fusion	Brillo et al. (2021)
*KLF15*	Kruppel like factor 15	Circadian clock transcription factor regulated by FoxOs that orchestrate the nitrogen metabolism by increasing amino acid catabolism and suppressing lipogenesis during fasting	Mattson et al. (2014) and Takeuchi et al. (2021)
*KLF9*	Kruppel like factor 9	Modulates cellular circadian clock and is overrepresented in muscle with CR	Kasai et al. (2020) and Knoedler et al. (2020)
*USP2*	Ubiquitin‐specific peptidase 2	A ubiquitin‐specific protease required for TNF‐induced NF‐kB (nuclear factor kB) signaling. Regulates the circadian clock by shuttling PER1 to the nuclei and by repressing the clock transcription factors CLOCK and ARNTL/BMAL1	Melzer et al. (2020) and Pouly et al. (2016), and Zhang et al. (2014)
*PRKACA*	Protein kinase cAMP‐activated catalytic subunit alpha	This has been found upregulated with aging and downregulated with CR in mice, involved in multiple cellular processes including glucose metabolism and cell division	Bareja et al. (2021)
*EFTUD2*	Elongation factor Tu GTP binding domain containing 2	GTPase component of the spliceosome complex, tends to be alternatively spliced with aging	Rodríguez et al. (2016)
*CERS3*	Ceramide synthase 3	Increases of ceramides in skeletal muscle with, probably in conjunction with CERS1 and CERS2	Obanda et al. (2015)
*PER1*	Period circadian regulator 1	A core clock gene that transcriptionally represses the negative limb of the feedback loop and interact with the CLOCK|NPAS2‐ARNTL/BMAL1|ARNTL2/BMAL2 heterodimer inhibiting its activity and thereby negatively regulating their own expression	Plank et al. (2012), Small et al. (2020), and Velingkaar et al. (2020)
*GCN1*	GCN1 activator of EIF2AK4	Acts as a positive activator of the EIF2AK4/GCN2 protein kinase activity in response to amino acid starvation. Participates in the repression of global protein synthesis and in gene‐specific mRNA translation activation, such as the transcriptional activator ATF4	Derisbourg et al. (2021)
*SNRNP200*	Small nuclear ribonucleoprotein U5 subunit 200	A core component of precatalytic, catalytic, and postcatalytic spliceosome complexes	Yang et al. (2021)
*AAR2*	AAR2 splicing factor	Component of the U5 snRNP complex required for spliceosome assembly and for pre‐mRNA splicing	Nakazawa et al. (1991)
*TBC1D14*	TBC1 domain family member 14	Plays a role in the regulation of starvation‐induced autophagosome formation. Contributes to the regulation of starvation‐induced autophagy	Longatti et al. (2012)
*PER2*	Period circadian regulator 2	A core clock gene that transcriptionally represses the negative limb of the feedback loop and interact with the CLOCK|NPAS2‐ARNTL/BMAL1|ARNTL2/BMAL2 heterodimer inhibiting its activity and thereby negatively regulating their own expression	Plank et al. (2012), Small et al. (2020), and Velingkaar et al. (2020)
*ELAVL1*	ELAV like RNA‐binding protein 1	RNA‐binding proteins that selectively bind AU‐rich elements (AREs) found in the 3′ untranslated regions of mRNAs and protect them from degradation. Resveratrol and CR actively regulate ELAVL1	Li et al. (2017)
*TINF2*	TERF1‐interacting nuclear factor 2	A component of the telomere nucleoprotein sheltering complex that protect telomeres from being exposed as DNA damage	Schmutz et al. (2020)
*GPX3*	Glutathione peroxidase 3	Protects cells and enzymes from oxidative damage, by catalyzing the reduction of hydrogen peroxide, lipid peroxides, and organic hydroperoxide, by glutathione	Chang et al. (2020)
*HSP90AA1*	Heat shock protein 90 alpha family class A member 1	Participate to the unfolded protein response with other chaperones (e.g., Hsp70)	Collino et al. (2013)
*SUV39H1*	SUV39H1 histone lysine methyltransferase	Interacts with the circadian target genes such as PER2 itself or PER1, contributes to the conversion of local chromatin to a heterochromatin‐like repressive state through H3 “Lys‐9” trimethylation. Upregulated by oxidative stress and CR in a SIRT1‐dependent manner	Bosch‐Presegué et al. (2011) and Mostoslavsky et al. (2010)
*BCL6*	B‐cell lymphoma 6	Inhibits the production of chemokines in macrophages in multiple tissues, and may also contribute to prevent cellular senescence in cardiac muscle cells	Altieri et al. (2012), Sommars et al. (2019), Yoo et al. (2017), and Yu et al. (2005)
*MEF2D*	Myocyte enhancer factor 2D	Plays diverse roles in the control of cell growth, survival and apoptosis via p38 MAPK signaling in muscle‐specific and/or growth factor‐related transcription. Also, involved in the circadian rhythm and regulation of sleep behavior as well as sleep and fasting cycle	Chen et al. (2015) and Mohawk et al. (2019)
*EIF1*	Eukaryotic translation initiation factor 1	Functionally similar to EIF41A, is upregulated by dietary restriction in obese women and enables translational initiation	Bjedov and Rallis (2020) and Nanda et al. (2009)
*CHERP*	Calcium homeostasis endoplasmic reticulum protein	Regulated alternative mRNA splicing events by interaction with U2 small nuclear ribonucleoproteins and U2 snRNP‐related proteins	Yamanaka et al. (2022)
*NR0B2*	Nuclear Receptor Subfamily 0 Group B Member 1	An orphan nuclear receptor that connects nutrient signaling with the circadian clock	Wu et al. (2016)
*SSRP1*	Structure‐specific recognition protein 1	Is a histone H2A/H2B chaperone proteins that plays a role in DNA single‐strand breaks repair	Gao et al. (2017)
*USP11*	Ubiquitin‐specific peptidase 11	P*lays an extremely important role in DNA damage repair*	Deng et al. (2018) and Liao et al. (2022)
*PCBP1*	Poly C binding protein 1	Has been found to regulate alternative splicing in many cancers	Huang et al. (2021)
(B) Under‐expressed genes in CR (*p* < 0.01)			
*IL1R1*	Interleukin 1 receptor type 1	Upregulated with aging mediates the biological activity of IL‐1β, and plays a role in type 2 diabetes	Grant and Dixit (2013) and Laberge et al. (2015)
*LURAP1L*	Leucine‐rich adaptor protein 1 like	Positively regulates I‐kappaB kinase/NF‐kB signaling	Jing et al. (2010)
*SKP2*	S‐phase kinase associated protein 2	SKP2 causes FOXO3 poly‐ubiquitination and proteasomal degradation; thus, the under‐expression of SKP2 protects FOXO3 from degradation	Cangemi et al. (2016) and Wang, Chan, et al. (2012)

*Note*: These genes were associated with CR previously or are known to play an important role in aging‐related biological mechanisms and/or muscle health.

### 2.3 Gene set enrichment analysis of CR

**FIGURE 3:**
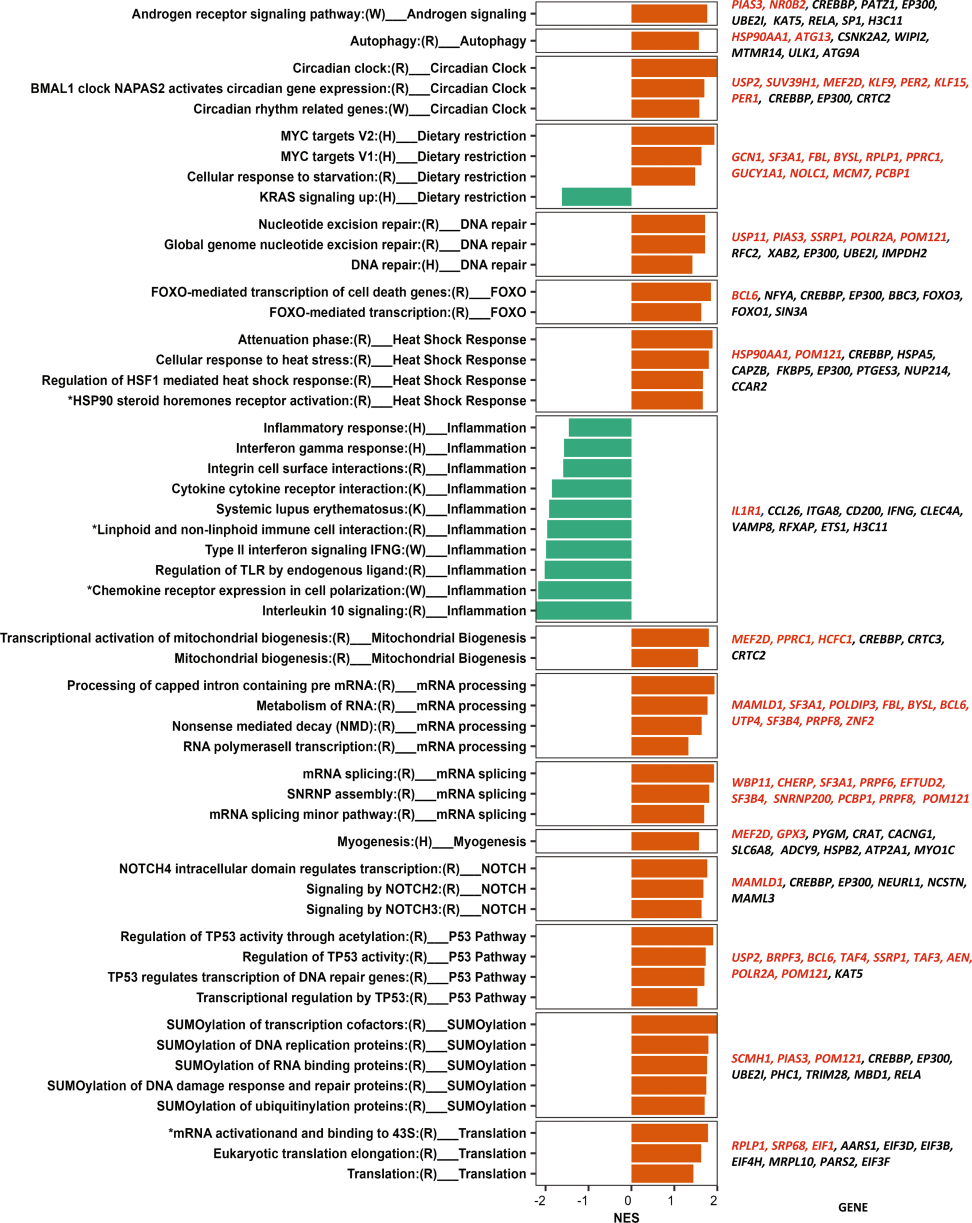
Selected significantly enriched pathways (*p*‐adj <0.05) obtained by ranked based gene set enrichment analysis. Genes were ranked by *p*‐value for the effect of CR compared to AL on differential expression change over time. The left column shows enriched pathways from four databases (H: Hallmark, R: Reactome, K: KEGG, and W: WikiPathways) followed by functional pathways domain. The middle column shows normalized enrichment score (NES) for each pathway (red upregulated pathways in CR and green downregulated pathways in CR). The last column reports up to 10 top significant genes (based on *p*‐value) in each domain, with red font indicating *p* < 0.01 and black font indicating 0.01 < *p* < 0.5. Star (*) symbol indicate that short name of the pathway was used for illustration purpose, full name can be seen in Table S4.

We performed rank‐based pathway enrichment analysis on the 16,450 protein coding genes using four major reference gene set collections, namely Hallmarks, Reactome, WikiPathways, and KEGG from the Molecular Signature Database MSigDB (v. 7.4; Subramanian et al., 2005; Methods). Altogether, we identified 140 enriched pathways (FDR adjusted *p*‐values <0.05), with the majority upregulated in CR compared to AL (the full list of pathways is reported in Table S4). Some of these pathways were redundant (same biological domain, overlapping leading genes) or not relevant in the context of the tissue and intervention examined (e.g., pathways related to G‐Couple receptors). A final subset of 53 pathways that appear to be meaningful for our analysis is shown in Figure 3. Confirming previous findings in model organisms and in keeping with the notion that CR enhances “anti‐aging” mechanisms, we found upregulation of androgen receptor signaling, autophagy, circadian rhythms, DNA repair, FOXO mediated transcription, heat shock response, mitochondrial biogenesis, mRNA processing and splicing, myogenesis, NOTCH signaling, P53 regulation (cellular senescence), SUMOylating and control of translation. Inflammation, together with KRAS signaling, were significantly downregulated in CR compared to AL (Figure 3).

Some differentially expressed genes and leading genes in the enriched functional pathways (Table 2) deserve particular mention (Table S5, Figure S4): Forkhead box O3 (*FOXO3*) is an ortholog of the DAF‐16 that mediates the pro‐longevity effects of DAF‐2 mutations in C. elegans and the effects of CR response in multiple species (Kenyon et al., 1993). Both *FOXO3* and *FOXO3B* genes were upregulated in CR. The function of *FOXO3* has been found associated with human longevity in multiple populations (Donlon et al., 2012; Jiang et al., 2019; Longo & Anderson, 2022; Mammucari et al., 2007; van der Horst & Burgering, 2007). Although *FOXO3B* is highly similar to *FOXO3*, its function and role in aging are unknown and may need further attention (Flachsbart et al., 2013; Santo & Paik, 2018). Heat shock protein family A (Hsp70) member 5 (*HSPA5*) participates in the unfolded protein response with other chaperones (e.g., *HSP90AA1*) and has been shown to be upregulated by CR in mouse hippocampus (Schafer et al., 2015). Cryptochrome circadian regulator 2 (*CRY2*), a core clock protein upregulated by CR, which also reduces its rhythmicity, reduced circadian rhythm upregulation (Patel et al., 2016; Velingkaar et al., 2021). Insulin receptor substrate 2 (*IRS2*) in skeletal muscle is associated with exercise and CR (Kang et al., 2023) and plays an important role in lipid metabolism in skeletal muscle, and longevity in mice (Kang et al., 2023; Masternak et al., 2005; Vega Magdaleno et al., 2022). SIK family kinase 3 (*SIK3*) is a serine–threonine kinase in the AMPK activated protein kinase family that a well‐known energy sensor. *SIK3* contributes to the circadian clock by impacting the stability of the *PER2* (Ng et al., 2019; Rijo‐Ferreira & Takahashi, 2019).

### 2.4 Gene expression changes induced by CR partially mediate preservation of muscle strength

To test the hypothesis that expression changes of specific genes mediate the beneficial effects of CR on mass‐adjusted muscle strength (both average power and peak torque in isokinetic dynamometry at 60 and 180°s^−1^ knee extension), we conducted regression analysis of CR effects on mass‐adjusted muscle strength. We focused on selected pathways identified as modified by CR (a total of 53 pathway as shown in Figure 3), and for each one of them, we calculated a pathway‐expression score (see Section 4). Regression models were run for different number of pathway selection (*k*). We first fitted regression models with assessing the effect of CR of change of mass‐adjusted muscle strength over the study follow‐up and then added to this basic model each of the individual pathway scores (when *k* = 1). Subsequently, we fitted models that included multiple pathways scores (2 ≤ *k* ≤ 4). Our goal was to assess whether the inclusion of such pathways scores (Equation 3) mediate the effect of CR on muscle quality assessed as the percent reduction of the beta coefficient for the CR effect compared to the basic model that did not include any pathways score (Equation 2). Only pathway combinations that explained at least 20% of the effect (reduction of the beta coefficient) were considered and reported as partial mediation.

**FIGURE 4:**
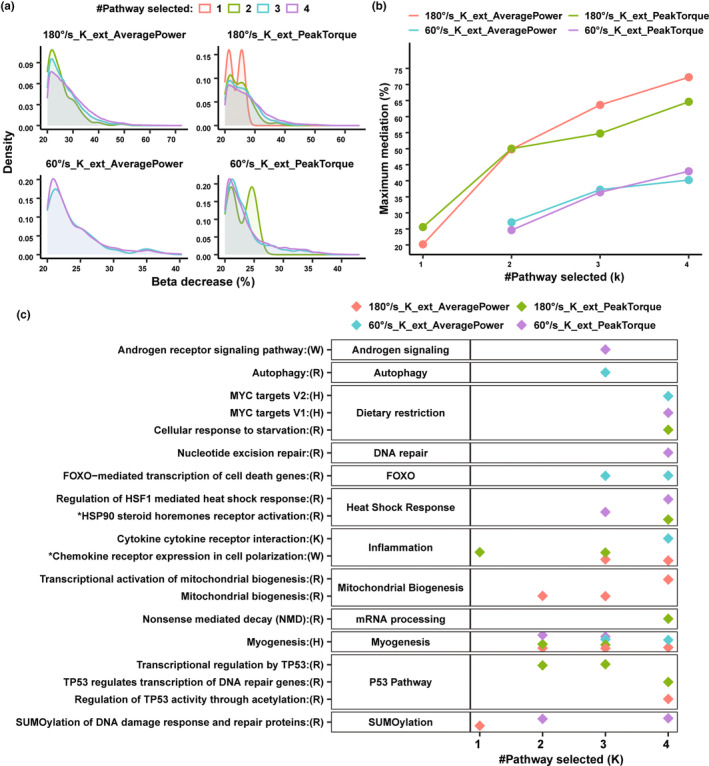
Pathways enriched for genes that differentially change expression over the follow‐up in CR compared to controls mediate the preservation of muscle quality. (a) Regression models analysis showing percent mediation (*x*‐axis, change in the beta coefficient for CR compared to AL) in models predicting changes in muscle quality. Only results for combination of pathways with mediation of 20% or more are shown from all combination of models with 1, 2, 3, and 4 pathways scores included, separately for each of the strength variables (colored). (b) A line with dot plot showing maximum percent mediation (%) as observed for specific number of pathways set selection k (1–4) in for regression model analyses. (c) Dot point represent specific set of pathways (for different *k*) that yield highest mediation considering pathway set from different pathways category for each of the strength variables. Star (*) symbol indicate that short name of the pathway was used for illustration purpose, full name can be seen in Table S4.

In the majority of the pathway combinations, we observe 25–30 beta percentage reduction compared to the basic model (Figure 4a). The maximum mediation effect for different number of pathway selection was seen for both strength variables in isokinetic dynamometry at 180°s^−1^ knee extension compared to isokinetic dynamometry at 60°s^−1^ knee extension (Figure 4b). The percent mediation increased for some pathways selection with the number of pathways scores included in the models (Figure 4b). Of the 53 pathways tested independently (*k* = 1) for muscle strength variable, we identified two unique pathways (SUMOylation of DNA damage response and repair proteins from SUMOylation category and Selective expression of chemokine receptor expression in cell polarization from Inflammation category) in which the size of the beta coefficient in the model for CR was reduced by 20% or more for strength peak torque and average power in isokinetic dynamometry at 180°s^−1^ knee extension (Figure 4c, Table S6). As many pathways are functionally similar, their average pathway scores as calculated (Methods) might be closer to each other because of gene set similarity. To avoid this problem, we highlighted top 3 pathway set for *k* ≥ 2 that were observed from different functional category and showed maximum mediation effect (Table S6). For example, average power in isokinetic task 180°s^−1^ knee extension when *k* = 2, we observed two pathways: Myogenesis and Mitochondrial biogenesis in multivariable model showed maximum mediation of ~50% compared to base model (Figure 4c). In spite of some differences across the four muscle strength variables (Figure 4c), there were many shared pathways that point to mechanisms that have been previously identified as mediating the effect of CR in animal models, including SUMOylation, Autophagy, DNA repair, FOXO, circadian rhythm, mitochondrial biogenesis, heat shock response, mRNA processing, and inflammation. These findings indicate that CR effects gene expression encompasses multiple biological pathways that jointly enhance muscle quality.

### 2.5 Differential changes of splicing variants analysis linear over time

Since previous studies suggested that CR exerts part of its beneficial effect by regulating the expression of alternative RNA splice forms, we took advantage of the extreme depth of our paired RNA‐Seq to investigate differential transcript expression and differential transcript usage over time between CR and AL, which can be used as proxy measures for splicing variants. To enhance the reliability of our analysis, we relied on two widely used analytical methods, Kallisto and RSEM (Bray et al., 2016; Li & Dewey, 2011; see Section 4). Because of the relatively small sample size, linear mixed model similar to those used for the differential gene expression analysis and a threshold *p* < 0.01 were used for statistical analyses. Comparing CR to AL, differential transcript expression analysis identified differential changes of 1043 splicing variants from 956 genes (Kallisto) and 675 splicing variants from 645 genes (RSEM); differential transcript usage analysis identified 1277 splicing variant changes from 1164 genes (Kallisto) and 1041 splicing variant changes from 922 genes (RSEM) at a significance threshold of *p* < 0.01 (Figure S5).

**FIGURE 5:**
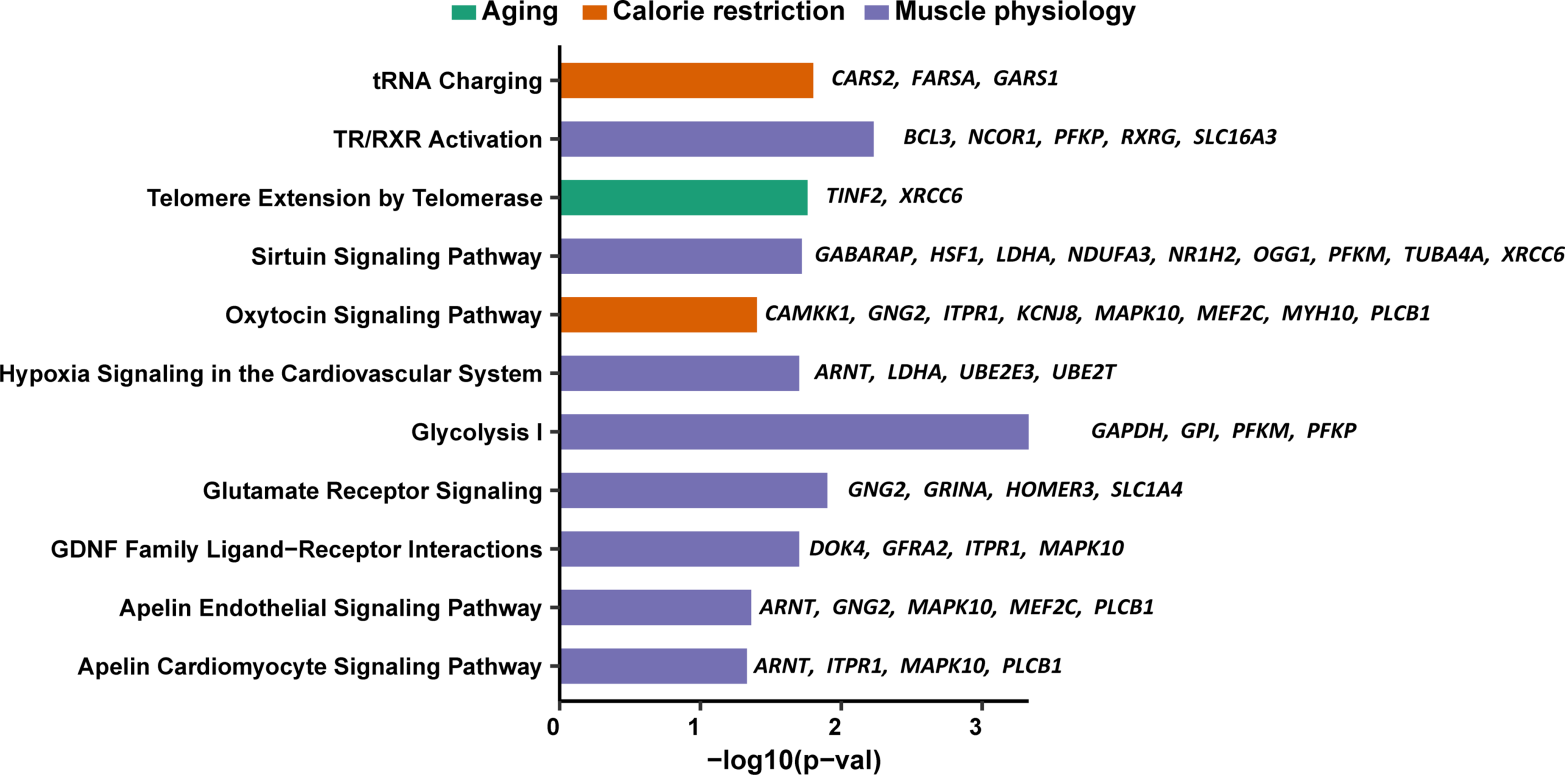
Significant selected pathways (*p* < 0.05) obtained through ingenuity pathway analysis (IPA) utilizing 328 genes for which at least one splicing variant was found to change significantly in CR compared to AL by differentially transcript expression and/or differentially transcript usage analysis. The left panel of the plot showing name of the significant pathways which were colored according to functional biological category (Aging, Calorie restriction and Muscle physiology). A list of leading genes in the right panel of each pathway (bar plot) were reported and supported mRNA splicing variants for each gene can be seen in Table S10.

We primarily focused on the 349 splicing variants (from 328 genes) that had been identified in both Kallisto and RSEM either by differential transcript expression (179 splicing variants from 174 genes; Table S7) or by differential transcript utilization (250 splicing variants from 235 genes; Table S8). To identify biological pathways associated with CR‐induced differential splicing, we performed ingenuity pathway analysis (IPA) on the set of 328 genes identified as described above (Table S9). The pathways identified as affected by the CALERIE™ intervention are aligned with protective effects of CR on muscle quality and attenuation of aging effects (Racette et al., 2017; Figure 5). Specifically, pathways enriched for alternative spliced forms included: Glycolysis—CR improves glucose utilization in muscle; TR/RXR Activation—important for muscle regeneration; Glutamate signaling—important role in muscle health; and oxytocin signaling pathway—necessary for muscle maintenance and regeneration (Elabd et al., 2014). GDNF Family Ligand‐Receptor Interaction family members, BDNF and GDNF, were increased in muscle with CR. The Apelin Cardiomyocyte Signaling Pathway and Apelin Endothelial Signaling Pathway, apelin, declined with CR (Yuzbashian et al., 2018). Particularly important, IPA analysis highlighted the Sirtuin signaling pathway, confirming that members of the Sirtuin family of histone deacetylases are biological mediators of CR (Bordone et al., 2007; Guarente, 2006). Finally, IPA analysis identified multiple pathways with known relationship to core mechanisms of aging biology, including Telomere Extension by Telomerase; a positive effect of CR on telomere length and telomerase activity has been well documented in rodents (Vera et al., 2013). Specific splicing variants along with variants transcribed from specific genes in these pathways are reported in Table S10.

**FIGURE 6:**
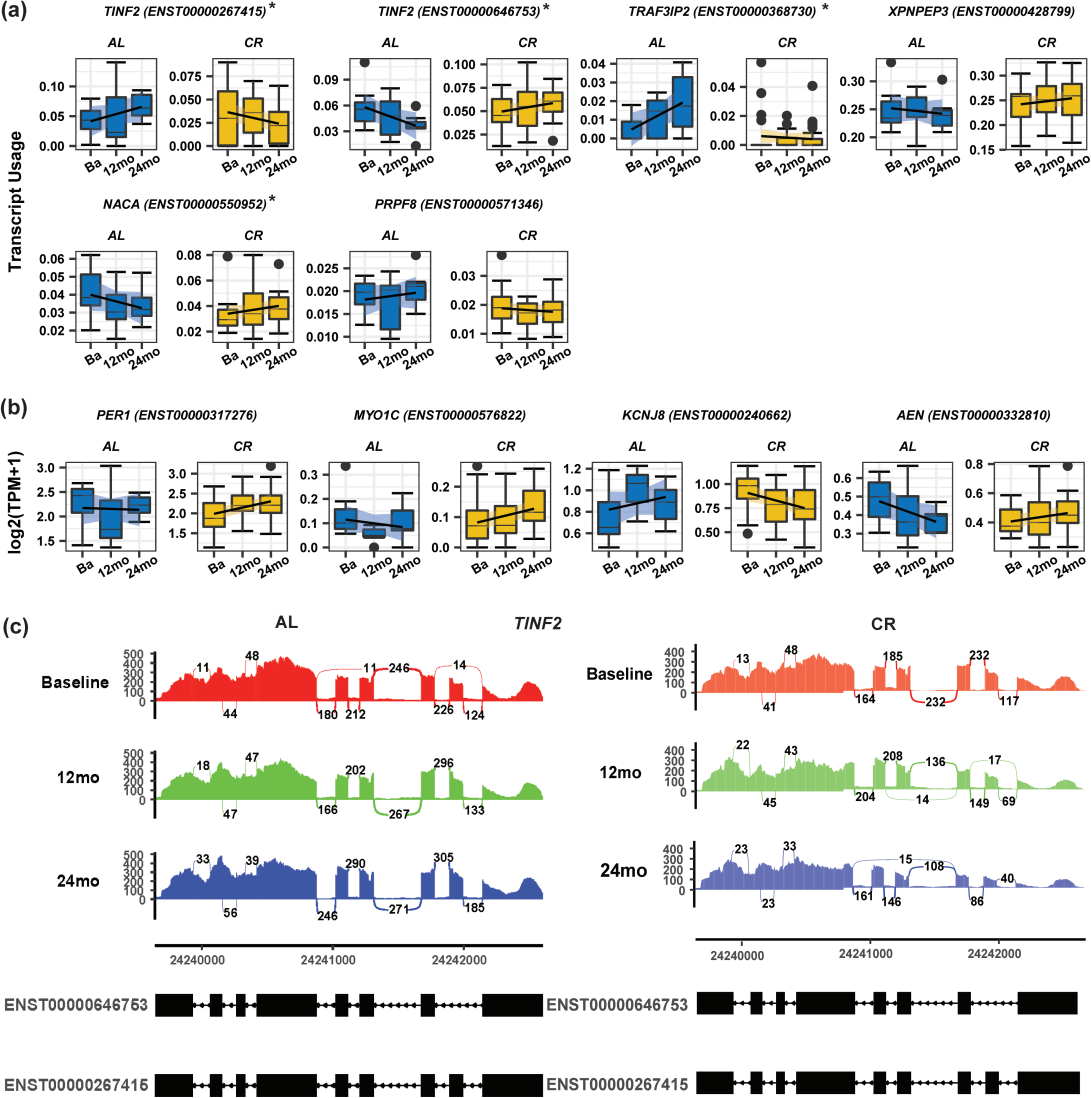
Differentially transcript expression change and/or differentially transcript usage changes of twelve mRNA splicing variants over time between CR and AL. Examples of splicing variants identified as showing significant differential expression changes/usage at *p* < 0.01 between CR and AL over time (baseline (Ba), 12‐month [12 mo], and 24‐month [24 mo]) in both Kallisto and RSEM analyses. Figure showed differentially expressed change supported transcripts in (a) and differentially usage change supported transcripts in (b) for completer group. Star (*) symbol in differentially usage supported transcripts was also supported in differentially expression analysis. Both differentially transcript expression (in log2(TPM + 1)) and differentially transcript usage (normalized by log2(TPM + 1) per RNA in 0–1 scale) changes were shown for Kallisto quantification approach and. (c) Sashimi plot (shown for one sample) representing junction count for two alternate splice variants of TINF2 separately for AL and CR group. Important junction is where the exon‐skipping event is occurring. For CR group, junction count value decreases over time at 12 and 24 months compared to baseline whereas as for AL group, value almost unchanged or little increasing in 12 and 24 months compared to baseline.

In further analyses, we focused on the 80 splice variants from 78 genes identified in both differential transcript expression and differential transcript utilization analyses (Table S11). Of these, 39 transcripts were protein coding, 13 were retained introns, 15 were processed transcripts, and 13 were part of the nonsense‐mediated decay surveillance category. Interestingly, 53 of the differentially expressed splice variants were mapped to genes that showed differential change in gene expression between CR and AL, suggesting that expression changes induced by CR involve both gene expression and the production of alternative spliced forms of mRNA. Of these variants, five variants were transcribed from the four genes that emerged from the differential gene expression analysis at the threshold of *p*‐ < 0.01 including: *TINF2* (Schmutz et al., 2020), *NACA* (Park et al., 2010), *PER1* (Plank et al., 2012; Small et al., 2020; Velingkaar et al., 2020), and *PRPF8* (Le Couteur et al., 2021; Rhoads et al., 2018; Zhang et al., 2019; Figure 6a,b). We also noticed variants transcribed from genes at a significance threshold *p* < 0.05 that are known to affect skeletal muscle or smooth muscle physiology (Figure 6a,b), such as *MYO1C* (myosin IC, that regulates glucose uptake in mouse skeletal muscle [Toyoda et al., 2011]); *KCNJ8* (potassium inwardly rectifying channel subfamily J member 8, that modulates brain vascular smooth muscle development and neurovascular coupling [Ando et al., 2022]); *TRAF3IP2* (TRAF3 interacting protein 2, an upstream regulator of various critical transcription factors, associated with vascular smooth muscle cell migration and proliferation [Mummidi et al., 2019]). Also, two important genes, *AEN* (apoptosis enhancing nuclease, Mediates p53‐induced apoptosis [Lee et al., 2005]), and *XPNPEP3* (X‐prolyl aminopeptidase 3, function as an adapter protein for *TNFRSF1B* [Inoue et al., 2015]), are related to apoptosis; notably, age‐related sarcopenia is characterized by apoptosis that is partially prevented by CR (Phillips & Leeuwenburgh, 2005; Wohlgemuth et al., 2010). Among the genes that code for these variants, differential splicing was confirmed for four genes (*PER1*, *MYO1C*, *KCNJ8*, *AEN*) by differential transcript expression analysis only, two genes (*PRPF8 and XPNPEP3*) by differential transcript usage analysis only, and three genes (*TINF2*, *NACA*, *TRAF3IP2*) were confirmed by both differential transcript expression and differential transcript usage.

Interestingly, our analyses also pointed to differential transcript expression and/or differential transcript usage for some splicing variants at *p* < 0.05 in genes showing no changes in global expression between CR and AL in the differential gene expression analysis (*p* > 0.05). For example, these included *ENST00000530661* and *ENST00000527328* transcribed from gene *HSF1* (heat shock One transcription factor 1; Raynes et al., 2013) and *ENST00000469865* transcribed from *MYH10* (myosin heavy chain 10). This finding suggests that while one splicing variant is overexpressed, others from the same gene are underexpressed (or vice versa) with possible changes in biological effect without detectable change in the differential gene expression analysis (Figure S6).

## 3 DISCUSSION

The preservation of muscle structure and function with aging is associated with healthy longevity and independence. The only intervention in humans proven to maintain muscle structure and function with aging is exercise (Egan & Zierath, 2013). However, in laboratory animals, including non‐human primates. calorie restriction (CR) also contributes to maintenance of healthy, functional muscle into late life (Rhoads et al., 2020). To test the potential for parallel benefits in humans, we conducted deep RNA sequencing of human skeletal muscle obtained from repeated biopsies over the course of the CALERIE™ trial, a two‐year randomized controlled trial of CR in healthy humans without obesity. We identified differential gene expression changes in CR participants compared to controls that point to biological pathways previously shown to be affected by CR in laboratory animals, including muscle repair and myogenesis and biological mechanisms of aging. For genes with differentially expressed and altered by CR intervention, we confirmed previous suggestions that alternative splicing is affected by CR. Our findings provide the first evidence that even moderate intensity CR preserves skeletal muscle health through molecular pathways previously associated with CR in animal models, such as circadian clock, mitochondrial health and biogenesis, proteostasis, alternative splicing, and inflammation.

Recent studies suggest that CR‐driven life extension is mediated by preserving the robustness and stability of the circadian rhythm, directly implicated in aging and age‐related chronic diseases (Eckel‐Mahan, 2023; Patel et al., 2016; Ulgherait et al., 2016; Zhao et al., 2019). Our study confirms that CR modulates the expression of many circadian genes in human skeletal muscle, including the upregulation of the BMAL1‐dependent core circadian genes *PER1* and *PER2*, as well as of the Sirt1‐dependent *USP2*, whose transcript represses the transcription factors CLOCK and ARNTL/BMAL1 by shuttling PER1 to the nuclei (Plank et al., 2012; Small et al., 2020; Velingkaar et al., 2020). Upregulation of PER1 and PER2 might prevent decreases in circadian amplitude of gene expression as reported in time‐restricted feeding with CR (Acosta‐Rodríguez et al., 2022).

KLF15 and KLF9 are two circadian clock transcription factors regulated by FOXO, a key metabolic regulator in the skeletal muscle (Kasai et al., 2020; Takeuchi et al., 2021). The modulation of FOXO by CR is crucial for the maintenance of metabolic homeostasis and removal of oxidative stress in skeletal muscle (Sanchez et al., 2014), as evidenced by increased insulin sensitivity and reduced inflammation, both under the control of FOXO3 signaling (Barthel et al., 2005). In recent studies, it has been also shown that FOXO3 drives inflammation in response to oxidative stress (Hwang et al., 2021). [Correction added on 03 November 2023, after first online publication: In the above sentence, reference citation Santo et al., 2023 has been replaced with Hwang et al., 2021.] The FOXO3A‐short mRNA (ENST00000540898.1), which is a FOXO3A transcriptional isoform, overexpressed in CR in our study (*p* < 0.05). However, studies also found that FOXO3A‐short suppresses glycolysis in skeletal muscle and downstream of PI3K/Akt signaling (Santo et al., 2023). [Correction added on 03 November 2023, after first online publication: In the above sentence, the following reference citations Bloedjes et al., 2023 and Hwang et al., 2021 has been replaced with Santo et al., 2023]. Recent studies showed that CR upregulated several genes, including *IRS2* which was upregulated by CR in this study (Kang et al., 2023). CR reduces insulin resistance and insulin levels, which may reduce the PI3K/AKT signaling and in turn increase the FOXO3A upregulation and transcriptional activity. Since some data in the literature on the effect of FOXO3A on glycolysis are controversial, we performed additional analyses mainly to evaluate the correlations between FOXO3 and its targeted genes as reported within the context of Akt signaling (Bloedjes et al., 2020, 2023), and considered genes also found significant in our differential gene expression analysis. Results showed mostly moderate or low correlations between FOXO3 and its targeted genes (Table S12). Given the important role of FOXO3 in aging and CR, the role of FOXO3 and glycolysis in human muscle deserves further in depth studies.

CR improves mitochondrial function and biogenesis leading to the attenuation of age‐related declines in mitochondrial function in skeletal muscle (Civitarese et al., 2007; Hancock et al., 2011; Kanzleiter et al., 2014; Lanza et al., 2012; López‐Lluch et al., 2006). Our study confirms the positive effects of CR on mitochondrial health by the changes induced in key mitochondrial genes *PKNOX1*, *PPRC1*, *PLD6*, and *PDP2*, suggesting increases in oxidative phosphorylation (Kanzleiter et al., 2014), mitochondrial biogenesis, mitochondrial fusion (Miller et al., 2019; Shi et al., 2021), with these leading to improvements in lipid metabolism (Baba et al., 2014; Choi et al., 2006; von Eyss et al., 2015), and enhancement in TCA cycle activity and mitochondrial respiration via pyruvate dehydrogenase complex activation (Baba et al., 2014; Bagherniya et al., 2018; Choi et al., 2006; Kanzleiter et al., 2014; Miller et al., 2019; Shi et al., 2021; von Eyss et al., 2015).

Autophagy and mitophagy remove toxic proteins and dysfunctional organelles, a mechanism essential for maintaining muscle health and preventing muscle loss and sarcopenia (Bagherniya et al., 2018; Korolchuk et al., 2010). We found that CR upregulated both processes and the SUMOylation (small ubiquitin‐like modifier) pathway, critically important in the refolding and/or clearance of damaged proteins, through the ubiquitin–proteasome pathway, chaperone‐mediated autophagy, and macroautophagy upon proteotoxic stress (Bekker‐Jensen & Mailand, 2011; Dou et al., 2011; Liebelt et al., 2019; Maduna et al., 2021).

In differential gene expression analysis, the top CR‐associated overexpressed gene was *TINF2*, a capping protein that protects telomeres. Whether CR affects telomere length is controversial, but recent reports suggest that *TINF2* has important extratelomeric functions (Pendergrass et al., 2001; Robin et al., 2020; Smith Jr et al., 2011). *TRF2* protein declines with aging in human skeletal muscle and its downregulation in vitro suppresses the mitochondrial SIRT3 protein causing mitochondrial dysfunction and oxidative stress. Both effects are reversed by restoring SIRT3 level. These data suggest that the CR‐induced upregulation of the TRF2‐SIRT3 axis elicits anti‐aging effects on muscle by improving mitochondrial function (Robin et al., 2020).

Calorie restriction may modulate stress pathways in response to DNA damage (Ke et al., 2020). The genes that were overexpressed in response to CR in our study included *SSRP1* and *USP11*, which are associated with a DNA‐repair pathway that might help cells to cope with oxidative damage and also enhance autophagy (Heydari et al., 2007; Ke et al., 2020; Parveen, 2021). There is evidence that CR reduces serum total and free testosterone, increases SHBG concentration in humans, in some studies, increases the ratio of estrogen to androgen (Cangemi et al., 2010; Słuczanowska‐Głąbowska et al., 2015).

CR lowers chronic inflammation (Mattison et al., 2017; Spadaro et al., 2022). In CALERIE, the moderate level of CR improved thymopoiesis by mobilizing the intrathymic ectopic fat (Spadaro et al., 2022). CR also leads to transcriptional changes in the adipose tissue implicated in pathways regulating mitochondrial bioenergetics, anti‐inflammatory responses, and lifespan (Spadaro et al., 2022). In studies in model organisms and in humans, CR reduces other “pro‐aging mechanisms” such as inflammation and coagulation (Lijnen et al., 2012; Starr et al., 2016). Our study highlighted the downregulation of several inflammation‐related pathways, including cytokine–cytokine receptor interaction, inflammatory response, and expression of *IL1R1*, whose transcript is involved in many cytokine‐induced immune and inflammatory pathways (Grant & Dixit, 2013).

Several lines of evidence suggest that the spliceosome functionality and alternative pre‐mRNA splicing are necessary for the effect of CR on longevity (Heintz et al., 2017; Tabrez et al., 2017). In our study, CR upregulated many spliceosomes protein‐ and RNA processing‐encoding genes and promoted splicing‐related pathways (as evidenced by the enrichment analysis [Table 2]). Some of the identified differentially expressed splice variants induced by CR come from the top 4 genes identified by differential gene expression, namely *TINF2* (Schmutz et al., 2020), *NACA* (Park et al., 2010), *PER1* (Plank et al., 2012; Small et al., 2020; Velingkaar et al., 2020), and *PRPF8* (Le Couteur et al., 2021; Rhoads et al., 2018; Zhang et al., 2019). Interestingly, many of the genes that demonstrated differential changes in spliced variants affect skeletal muscle or smooth muscle physiology, notably the *MYO1C‐*encoding transcript which regulates glucose uptake in mouse skeletal muscle (Toyoda et al., 2011). Based on the enrichment analysis, focused on differentially expressed transcripts, glycolysis, tRNA charging pathway, and the sirtuin pathway were the top biological mechanisms targeted by CR. These results are consistent with the notion that CR improves glucose utilization in muscle and protein translation (García‐Flores et al., 2021). The Sirtuin pathway emerged from our enrichment analysis of differentially expressed splicing transcripts and from the SIRT1 association with CR‐mediated differential expression of circadian genes. The ability of SIRT1 to directly interact and regulate a host of transcription factors (e.g., PPARγ, PGC1α, p53, and FOXO) supports the concept that the beneficial metabolic effects of CR are mediated by the Sirtuin system (Bordone et al., 2007; Guarente, 2006), but this effect is mediated at least in part by the differential expression of different splicing variants.

In conclusion, our study contributes to the knowledge about the mechanisms of the health benefits of CR by elucidating a widespread skeletal muscle transcriptional impact of a moderate and humanly attainable CR intervention over a 2‐year period that mediates, at least in part, the preservation of muscle function. The biological mechanisms identified are the same as those affected by CR in animal models: the Sirtuin pathway, promotion of mitochondrial biogenesis and mitochondrial health, enhancement of autophagy and circadian rhythm, reduction of inflammation, and upregulation of alternative splicing. We used state‐of‐the‐art RNA‐Seq technology, with a depth (387–618 million paired reads) greater than any other previous studies of muscle in humans. Because of the data quality, rank‐based GSEA analysis was employed to leverage all the available information. Through DGE and GSEA analyses, several genes were identified that have not been previously discussed in the context of muscle effects of CR (Table 2 and Table S4), notably changes in biological pathways that mediate the positive effect of CR on muscle quality. The contribution of alternative splicing in regulating key biological mechanisms targeted by CR is noteworthy. Taken together, this study reveals major molecular and cellular mechanisms triggered by a moderate reduction (~12%) of caloric intake in human skeletal muscle, and provides compelling evidence for a role of metabolism and alternative splicing, not only in skeletal muscle function, but also in the mechanisms of CR.

### 3.1 Limitations of the study

Our study was limited by the relatively small sample size. We could not replicate our findings in an independent cohort of participants, simply because no similar study has ever been performed in humans. Furthermore, while meaningful and significant transcriptomic changes were observed, they have not yet been validated at the proteomic level. It is crucial to recognize that discrepancies between the different omics levels of molecular organization are common and should be expected. In addition, our analysis excluded 17.5% of protein‐coding genes (as mentioned in the Results section) because of their low count in many samples. Pathway analysis with these genes (using g:Profiler) identified some important pathways, such as steroid biosynthesis, which is missing in this report (Table S13). However, given the very high depth of sequencing used in this study (range 387–617 million reads), which was higher than any previous transcriptomic studies in skeletal muscle, we suspect that most of these genes are not highly expressed in skeletal muscle. Despite these limitations, most of our findings confirm previous reports on the effects of CR in animal models and show positive effects of moderate CR in humans. These results support conducting a subsequent randomized controlled trial that builds on the results of CALERIE and includes a larger and more diverse population over a wider age range.

## 4 METHODS

### 4.1 Skeletal muscle biopsy study population

A detailed description of the CALERIE study design, participants characteristics, and intervention have been reported elsewhere (Ravussin et al., 2015), and the experimental design for whole CALERIE phase 2 participants can be found in CALERIE bio repository (https://calerie.duke.edu/sites/default/files/2022‐05/phase2_protocol.pdf). A total of 220 participants were randomly assigned to the CR (calorie restriction) group with a target of 25% below baseline level or to an AL (“ad libitum”) control group. Participants on CR received an intensive behavioral intervention administered by psychologists and nutritionists aimed at enhancing adherence to the suggested diet. Calorie intake was objectively monitored by assessing total energy expenditure (TEE) assessed by the doubly labeled water method (Lifson & McClintock, 1966). A total of 90 eligible individuals (a subset of whole CALERIE participants) participated in the muscle biopsy procedure yielding a total of 162 muscle biopsies over the 2‐year study period (baseline, 12‐, 24‐month) distributed between the CR (*n* = 57) and AL (*n* = 33) groups with muscle biopsies were missing two participants in the CR group who had follow‐up but not a baseline and for the 12‐ and 24‐month timepoints for many who had a muscle biopsies at baseline (Table S1).

### 4.2 Leg lean mass and muscle strength measure

Leg lean mass was assessed by dual‐energy x‐ray absorptiometry (DXA; Hologic Inc.), measured in kilograms (kg), and details measure are reported in (Racette et al., 2017). Leg lean mass is calculated by leg lean mass after subtraction of the bone marrow (i.e., leg lean mass = fat free mass [leg] − bone marrow contains [leg]) and considered as an approximation of muscle mass in our analysis. Similarly, the details of the muscle strength measured were reported in (Racette et al., 2017). Here, we utilized two muscle strength variables (absolute peak torque and average power) for knee extension in isokinetic dynamometry at 60 and 180°s^−1^, measured in newton meter (Nm). For purposes of analyses, both muscle mass and strength variable, absolute value for the right and left legs were averaged.

### 4.3 Sample *acquisition*, preparation and next generation sequencing

#### 4.3.1 Muscle biopsies

Skeletal muscle biopsies of the vastus lateralis (VL) muscle were obtained using a 25‐gauge, 2 inch needle as described in Bergstrom (Bergström, 1975). Briefly, the biopsy site in fasting participants (12 h) was treated with local anesthesia. A small incision was made using a scalpel, and the biopsy needle was inserted. Approximately 50–100 mg of muscle tissue was extracted and placed in a cryovial and immediately flash frozen in liquid nitrogen. Samples were stored at −80°C until processing.

#### 4.3.2 RNA extraction

Sections of flash frozen VL skeletal muscle (~50 mg) were homogenized using a bead‐based Qiagen TissueLyser II in l mL TRIzol™ Reagent (Invitrogen™ ThermoFisher #15596026). Following homogenization, RNA was extracted using a Qiagen RNeasy Mini Kit (Qiagen #74104) and stored at −80°C until mRNA sequencing.

#### 4.3.3 mRNA sequencing

Illumina libraries were generated with the TruSeq Stranded Total RNA Library Preparation Kit. RNA was sequenced using the *Illumina NovaSeq 6000* sequencing system with paired‐end reads. The samples yielded 387–618 million pass filter reads with more than 86% of bases above the quality score of Q30.

### 4.4 Filtering, alignment and genome annotation

The quality of reads in fastq RNA‐Seq files were initially assessed using *FastQC* tool (v. 0.11.5; https://www.bioinformatics.babraham.ac.uk/projects/fastqc/), Preseq (v. 2.0.3; Daley & Smith, 2013), Picard tools (v. 2.17.11; https://broadinstitute.github.io/picard/), and RSeQC (v. 2.6.4; Wang, Wang, & Li, 2012). Reads were trimmed using *bbduk* (from *bbtools* package; https://jgi.doe.gov/data‐and‐tools/software‐tools/bbtools/; Bushnell et al., 2017). Following trimming, cleaned reads were examined one more time using *FastQC*. Next, cleaned fastq files, along with reference human genome 38 and Ensembl annotation v104, were used as input for STAR (version 2.7.10a), a splice‐aware aligner implemented with a novel algorithm for aligning high‐throughput long and short RNA‐Seq data to a reference genome (Dobin et al., 2013). The STAR aligner was run with the—*quantmode TranscriptomeSam* parameter to also generate transcriptome BAM files, and was used for RSEM analysis. Genome BAM files were sorted and indexed using samtools. Finally, genome BAM files were used as input for f*eatureCounts* from the Rsubread package (version 2.0.1; Liao et al., 2014), a suitable program for counting reads for various genomic features such as genes. To generate expression data for splicing analysis, transcriptome BAM files were used for running RSEM (Li & Dewey, 2011), and cleaned fastq files were used for running Kallisto (Bray et al., 2016).

### 4.5 Differential gene expression analysis model

Since this study focused on protein‐coding transcripts, all non‐coding RNAs were excluded. Also excluded were RNAs with read count = 0, in 50% or more samples in any of the study subgroups (baseline, 12‐, 24‐month) of CR or AL (*n* = 3450). Then, the raw read count of the remaining 16,450 RNAs was normalized and converted to log2 transformed counts per million (CPM), that is, log2(CPM), using the edgeR package implemented in R (Robinson et al., 2010). Next, for each RNA with log2(CPM), linear mixed effect models (LMM) were built using the lme4 package implemented in R (Bates et al., 2015) to estimate associations of randomization group (CR vs. AL) with baseline RNA expression levels and RNA expression trajectories over time, adjusting for baseline age, baseline bmi, sex, race, and sequence batch in the mean model, and utilizing random intercepts in the variance model. A benefit of using LMMs in this study is their operation under an inherent Missing At Random (MAR) assumption (Griswold et al., 2021). MAR was supported from both similarities in characteristics between those dropping out versus those completing the study, and also from the reasons for missingness, which were primarily due to biopsy pain avoidance and not due to muscle outcomes of interest reported here. RNA expression trajectories (linear changes over time) for each randomization group, and differences between these groups in their trajectories (estimated by the interaction term between randomization group and linear time‐years) were estimated in our primary LMMs as shown in Equation 1. We then extracted *p*‐values (default t‐tests use Satterthwaite's method [*“lmerModLmerTest”*]) and *β*‐values (represent trajectory slope difference in CR compared to AL) from LMMs that we considered likely to identify the most significant protein‐coding transcripts that changed differentially between CR and AL over time. Ranking of protein‐coding transcripts using extracted *p*‐values with signs of *β*‐values from LMM models (linear time) was used in enrichment analyses (discussed below).

For expression results, we also incorporated additional information using similar LMM models but replaced the linear time terms with visit indicator functions (non‐linear time) to estimate differentially expressed transcripts at the specific 12‐ and 24‐month visits between the CR and AL groups (Equation 1). Similarly, we also extracted *p*‐values and *β*‐values from LMMs for 12‐ and 24‐month expression between the CR and AL groups. Both approaches can be described via the equation:

(1) $$\begin{array}{c} {\text{GeneExp}}_{i}∼\beta_{0}+\beta_{1}\left(\text{Group}\times \text{Time}\right)+\beta_{2}\text{Group}+\beta_{3}\text{Time}\\ \;+\beta_{4}\mathrm{Age}+\beta_{5}\mathrm{Bmi}+\beta_{6}\mathrm{Sex}+\beta_{7}\text{Batch}+\beta_{8}\text{Race}+u_{i}+\varepsilon_{i} \end{array}$$

where $\beta_{1}$ is the main effect (slope) that represents the trajectory difference between CR and AL groups, time is either continuous (years from baseline) or discrete (baseline, 12‐, and 24‐month visits) and $u_{i}$ is a random intercept to account for participant differences. Quantitatively, significant protein‐coding transcript results were compared for both approaches.

We used a threshold of *p* < 0.05 (unadjusted) for differential gene expression analysis. The top most significant genes that relate to influencing biological mechanisms through calorie restriction were considered for further characterization and exploration through literature review, and also using GeneCards (Stelzer et al., 2016). The package ggplot2 implemented in R (Wickham et al., 2016) along with various other R packages were used for generating various plots and linear smooth trajectories for selected transcripts.

### 4.6 Enrichment analysis

Following best practice procedures for pathway enrichment analysis on ranked gene lists spanning all or most of the genes in the genome, we performed Gene Set Enrichment Analysis (GSEA), a threshold‐free method that analyzes all measured genes without prior gene filtering (Reimand et al., 2019). GSEA was implemented using package *fgsea* (v.1.20.0; Korotkevich et al., 2021), [doi:10.1101/060012] and reference gene set collections Hallmarks (50 gene sets), Canonical Pathways including Reactome (1615 gene sets), WikiPathways (664 gene sets), and KEGG (186 gene sets) from the Molecular Signatures Database (MSigDB v.7.4; Subramanian et al., 2005). The rank metric was *r* = −log_10_(*p*‐value)*sign(beta), which captures both the significance of the differential gene expression and the direction of change. A customized implementation in R was developed in‐house to add robustness to the GSEA analysis; since GSEA's enrichment estimates (and statistical significance) are stochastic, our software embeds GSEA in a Monte Carlo algorithm that performs 1000 iterations and chooses significant pathways based on the total statistical ensemble. We report only pathways that appeared significant (based on the adjusted *p* < 0.05) in more than 80% of the iterations. We also used g:Profiler (https://biit.cs.ut.ee/gprofiler/gost) for enrichment analysis with selected gene list (Raudvere et al., 2019).

### 4.7 Mediation analysis model

We performed a mediation analysis to test the hypothesis that after adjusting for changes in muscle mass (leg lean mass), gene expression changes in muscle associated with CR compared to AL account for the changes in muscle strength. Of the original study population of 90 participants with 162 samples used for differential gene expression analysis, for this analysis we used data from 28 participants from the CR group and 16 participants from the AL group who had data at baseline and at least one follow‐up visit (of note, we originally had 17 participants for the AL group, but one participant with incomplete muscle mass data was excluded). As proxy measures of muscle mass, we calculated change in muscle mass (DeltaMass) between baseline and 24 months or baseline and 12 months if the 24‐month data were missing. Similarly, we calculated changes of muscle strength (DeltaStrength) for both average power and peak torque in isokinetic dynamometry at 60 and 180°s^−1^ knee extension between baseline and one follow‐up visit.

We fitted a linear regression model for average change of muscle strength (DeltaStrength) controlling for change of muscle mass (DeltaMass) and sex, as follows (Equation 2):

(2) $${\text{DeltaStrength}}_{i}\sim \beta_{0}+\beta_{1}\text{Group}+\beta_{2}\text{DeltaMass}+\beta_{3}\mathrm{Sex}+\varepsilon_{i}$$

where $\beta_{1}$ is the main effect that represents the average difference of DeltaStrength between CR and AL groups adjusted for DeltaMass and sex.

For mediation analysis, we computed pathway‐specific scores as follows. First, for all leading genes of each pathway, we normalized log2(CPM) gene expression values (*z*‐score) across all samples. Then for each sample, we calculated a pathway‐specific score as the mean gene expression of the leading genes in that pathway. Finally, we computed “pathway‐specific change scores” (DeltaPathway) between baseline and 12 months/24 months using the same approach as discussed above for muscle mass and muscle strength variable. Muscle mass, muscle strength, and pathway scores were transformed to standard normal (Z‐transform). Pathway‐specific change scores were included in regression models estimating the effect of CR versus AL on change in muscle strength after adjusting for change in muscle mass and sex (Equation 3). A meaningful “mediating” effect (partial mediation) was inferred when the pathway inserted in the model reduced by 20% or more the size of the beta coefficient for CR. Regression models were executed started with individual pathway (*k* = 1, simple linear regression model) to see the effect of individual pathway mediation effect and run with combinations of multiple pathways (*k* ≥ 2, multivariable regression model) to see the cumulative mediation effect on different pathways that globally mediate the effect of CR on muscle quality.

(3) $$\begin{array}{c} {\text{DeltaStrength}}_{i}∼\beta_{0}+\beta_{1}{\text{Group}}_{i}+\beta_{2}\text{DeltaMass}+\beta_{3}\mathrm{Sex}\\ \;+\beta_{j1}{\text{DeltaPathway}}_{1}+\beta_{j2}{\text{DeltaPathway}}_{2}\ldots \beta_{\mathrm{jk}}{\text{DeltaPathway}}_{k}+\varepsilon_{i} \end{array}$$

where $\beta_{1}$ is the main effect that represents the average difference of DeltaStrength between CR and AL groups, adjusted for DeltaMass, Sex, and DeltaPathway.

### 4.8 Differential splicing variant analysis model

Next, we investigated alternate splicing (AS) variants of all protein‐coding RNAs. We used two transcripts count quantification softwares, namely Kallisto (Bray et al., 2016) and RSEM (Li & Dewey, 2011), that provide transcripts per kilobase million (TPM) values of 19,950 RNAs. In both, we exclude transcripts that did not show transcripts per million (TPM) value > 0 in at least 2 samples in all study subgroups (baseline/12‐month/24‐month) for both the CR and AL groups. Also, RNAs associated with only one transcript were removed. Finally, 102,540 transcripts (or splicing variants) associated with 14,563 protein‐coding genes in both Kallisto and RSEM were considered for downstream analyses. We obtained TPM values from both the methods then converted them to log2‐normalized TPM, that is, log2(TPM + 1), which is considered for differential transcript expression analysis. Further, instead of raw count values, sample wise for each RNA, we also calculated normalized log2(TPM + 1) values taking all transcripts (0–1 scale), which is considered for differentially transcript usage analysis. In both approaches, we then used linear mixed effect models (similar to Equation 1), to determine both differential transcript expression and differential transcript usage differential change over time between CR and AL. Similarly, *p*‐values and *β*‐values were also extracted, and *p* < 0.01 (unadjusted *p* < 0.01) was considered the threshold for significance for candidate splice variants. We also performed enrichment analysis using ingenuity pathway analysis (IPA; software v01‐21‐03)(Krämer et al., 2014) taking all genes with at least one identified mRNA splicing variant that showed differential transcript expression and/or differential transcript usage changes significantly between CR and AL.

The detailed summary (commercial assays, data, experimental models, software tools, and algorithms used in this study) is provided in Table S14.

## AUTHOR CONTRIBUTIONS

All authors participated in different aspects of the study. J.K.D., D.W.B., and L.F. designed the study. J.K.D. performed all bioinformatics, computational and statistical analysis. N.B. and S.D. supported the bioinformatics work. J.C., D.W.B., and M.G. and supported in computational and statistical analysis design. M.O. generated muscle biopsy data. J.C., R.d.C., D.L.C., S.K.D., K.M.H., V.B.K., W.E.K., C.K.M., S.B.R., L.M.R., B.S., and D.W.B. critically reviewed the manuscript and provided for important intellectual content. J.K.D. and L.F. wrote the original manuscript. All authors reviewed the manuscript and approved for submission. L.F. supervised the whole study.

## CONFLICT OF INTEREST STATEMENT

None declared.

## Supporting information


**Figure S1.** Quantitative results for differentially expressed protein coding RNAs supported in differential gene expression analysis between CR and AL in the LMM (between Baseline & 12‐month (12 mo) and Baseline & 24 month (24 mo) and linear assumption over time).
**Figure S2.** Liner smooth trajectories of supported differentially expressed all significant genes obtained through differential gene expression analysis between CR and AL in the LMM (linear over time) at *p* < 0.01 as discussed in Table 2.
**Figure S3.** Pearson correlation (*p*‐value) between gene expressions (those are reported in Table 2) and muscle strength variables (peak torque and average power in the isokinetic task at 60 and 180°s^−1^) at Baseline.
**Figure S4.** Liner smooth trajectories of supported differentially expressed significant genes obtained through differential gene expression analysis between CR and AL in the LMM (linear over time) at 0.01 < *p* < 0.05 as discussed in the manuscript text.
**Figure S5.** Quantification of shared transcripts that were supported differentially changes both in Kallisto and RSEM approaches obtained through differential transcript expression and/or differential transcript usage between CR and AL in the LMM (linear over time) at *p* < 0.01.
**Figure S6.** Significant splicing variants that were differentially changed by both in differential transcript expression and differential transcript usage analysis between CR and AL in the LMM (liner over time) at *p* < 0.01 transcribed from genes that did not find significant (*p* > 0.05) in differential gene expression analysis.

**Table S1.** Characteristics of study participated in muscle biopsy program.

**Table S2.** Beta, slope, *p*‐value for all supported differentially expressed significant genes identified through differential gene expression analysis between CR and AL in the LMM (linear over time) at *p* < 0.01.

**Table S3.** Beta, slope, *p*‐value for supported differentially expressed significant genes identified through differential gene expression analysis between CR and AL in the LMM at *p* < 0.01 discussed in Table 2.

**Table S4.** List of identified all pathways for four reference databases (Hallmark, Reactome, KEGG, WikiPathways) obtained through GSEA analysis at *p*‐adj(median) < 0.05.

**Table S5.** Beta, slope, *p*‐value for supported differentially expressed significant genes through differential gene expression analysis between CR and AL in the LMM at 0.01 < *p* < 0.05 as discussed in the manuscript.

**Table S6.** Results of regression models (maximum of top 3 set of pathways) without and with pathway score adjusted identified as potential “mediators” with beta decrease 20% or more in at least one of the muscle strength variables.

**Table S7.** Beta, slope, *p*‐value for 179 significant differentially change splicing variants supported by Kallisto and RSEM approaches through differential transcript expression analysis between CR and AL in the LMM (linear over time) at *p* < 0.01.

**Table S8.** Beta, slope, *p*‐value for 250 significant differentially change splicing variants supported by Kallisto and RSEM approaches through differential transcript usage analysis between CR and AL in the LMM (linear over time) at *p* < 0.01.

**Table S9.** Identified significant pathways obtained through Ingenuity Pathway Analysis (IPA) at *p* < 0.05 taking for 328 genes (as mentioned in Tables S7 and S8) for which at one splicing variant transcript was found significant in differential transcript expression and/or differential transcript usage analysis between CR and AL in the LMM (linear over time).

**Table S10.** Listed of splicing variants that were found significant at *p* < 0.01 either in differential transcript expression or differential transcript usage analysis between CR and AL in the LMM (linear time) that were observed in selected pathways (as shown in Figure 5).

**Table S11.** Beta, slope, *p*‐value for 80 significant differentially change splicing variant that were supported by both Kallisto and RSEM approaches both in differential transcript expression and differential transcript usage analysis between CR and AL in the LMM (linear over time) at *p* < 0.01.

**Table S12.** Results of Pearson correlations analysis with gene expression values from this study between FOXO3 gene and its FOXO3 targeted genes those are described in Bloedjes et al., 2023 and 2020 (MM1.S FOXO3 knockout clones and XG‐3 FOXO3 knockout clones) also found significant in differential gene expression analysis with *p* < 0.05.

**Table S13.** List of identified all pathways (Reactome, KEGG, WikiPathways) at *p*‐value<0.05 (adjusted) obtained through g:Profiler (default setting) using excluded (due to their low count in many samples) protein coding genes.

**Table S14.** Key resources: commercial assays, data, experimental models, software tools and algorithms used in this study.

## Data Availability

All data and materials that support the findings of this study are available within the manuscript and supplemental information. RNA‐seq data were deposited in CALERIE bio repository that they can be obtained by request from the CALERIE bio repository. Any information required to reanalyze the data reported in this paper is available from the lead contact upon request.
