# Supplementary material for: Calorie restriction modulates the transcription of genes related to stress response and longevity in human muscle: The CALERIE study
Source: Aging Cell. 2023 Oct 12;22(12):e13963. doi: 10.1111/acel.13963 (PMC10726900; doi:10.1111/acel.13963)
Supplement: Supplementary file 1 — Figure S1. Quantitative results for differentially expressed protein coding RNAs supported in differential gene expression analysis between CR and AL in the LMM (between Baseline & 12‐month (12 mo) and Baseline & 24 month (24 mo) and linear assumption over time). Figure S2. Liner smooth trajectories of supported differentially expressed all significant genes obtained through differential gene expression analysis between CR and AL in the LMM (linear over time) at p < 0.01 as discussed in Table 2. Figure S3. Pearson correlation (p‐value) between gene expressions (those are reported in Table 2) and muscle strength variables (peak torque and average power in the isokinetic task at 60 and 180°s−1) at Baseline. Figure S4. Liner smooth trajectories of supported differentially expressed significant genes obtained through differential gene expression analysis between CR and AL in the LMM (linear over time) at 0.01 < p < 0.05 as discussed in the manuscript text. Figure S5. Quantification of shared transcripts that were supported differentially changes both in Kallisto and RSEM approaches obtained through differential transcript expression and/or differential transcript usage between CR and AL in the LMM (linear over time) at p < 0.01. Figure S6. Significant splicing variants that were differentially changed by both in differential transcript expression and differential transcript usage analysis between CR and AL in the LMM (liner over time) at p < 0.01 transcribed from genes that did not find significant (p > 0.05) in differential gene expression analysis. [file ACEL-22-e13963-s002.pptx]

## Slide 1
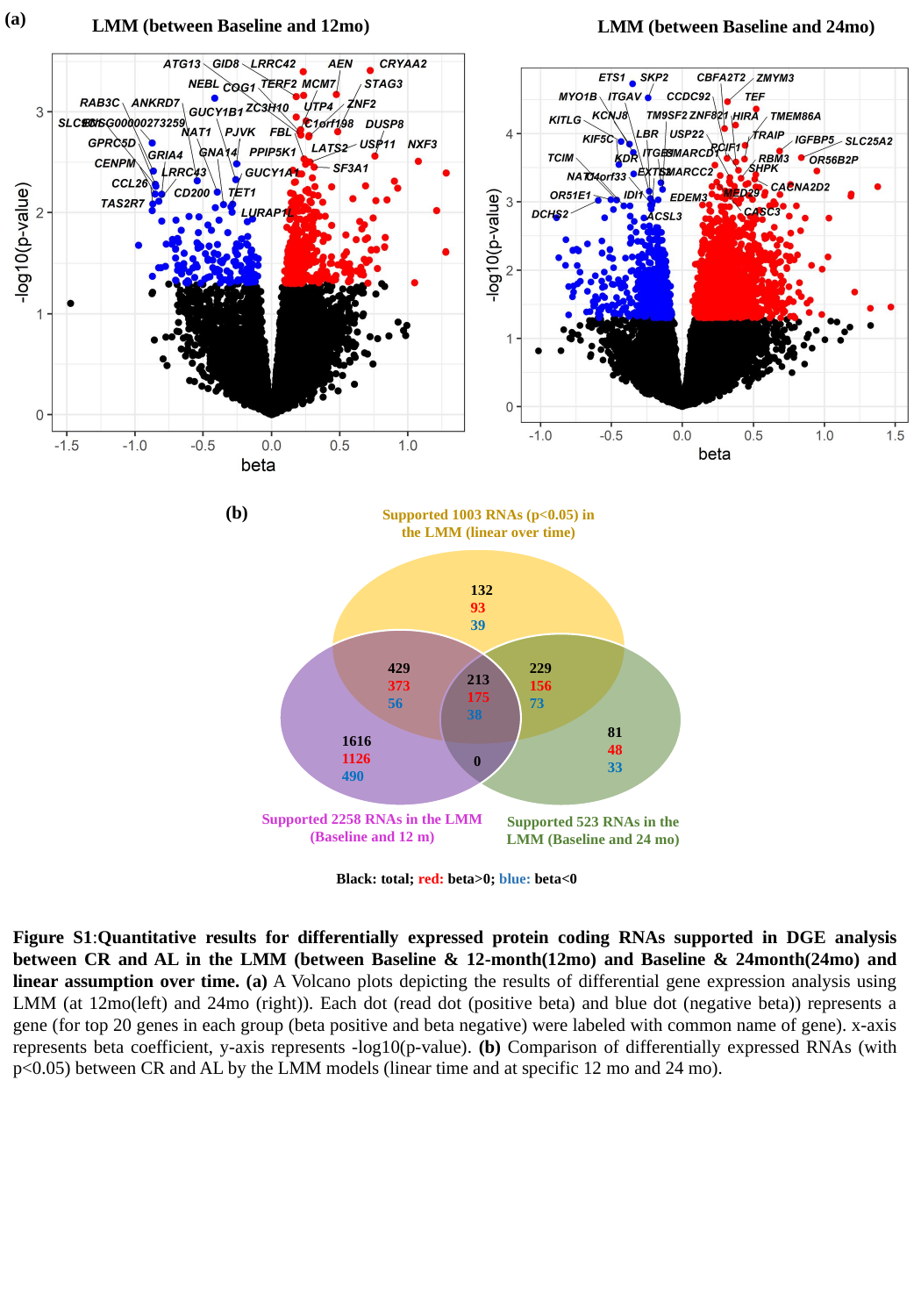

(a)
LMM (between Baseline and 12mo)
LMM (between Baseline and 24mo)
(b)
Supported 1003 RNAs (p<0.05) in the LMM (linear over time)
229
156
73
429
373
56
213
175
38
Supported 2258 RNAs in the LMM (Baseline and 12 m)
Supported 523 RNAs in the LMM (Baseline and 24 mo)
132
93
39
Black: total; red: beta>0; blue: beta<0
81
48
33
1616
1126
490
0
Figure S1:Quantitative results for differentially expressed protein coding RNAs supported in DGE analysis between CR and AL in the LMM (between Baseline & 12-month(12mo) and Baseline & 24month(24mo) and linear assumption over time. (a) A Volcano plots depicting the results of differential gene expression analysis using LMM (at 12mo(left) and 24mo (right)). Each dot (read dot (positive beta) and blue dot (negative beta)) represents a gene (for top 20 genes in each group (beta positive and beta negative) were labeled with common name of gene). x-axis represents beta coefficient, y-axis represents -log10(p-value). (b) Comparison of differentially expressed RNAs (with p<0.05) between CR and AL by the LMM models (linear time and at specific 12 mo and 24 mo).

## Slide 2
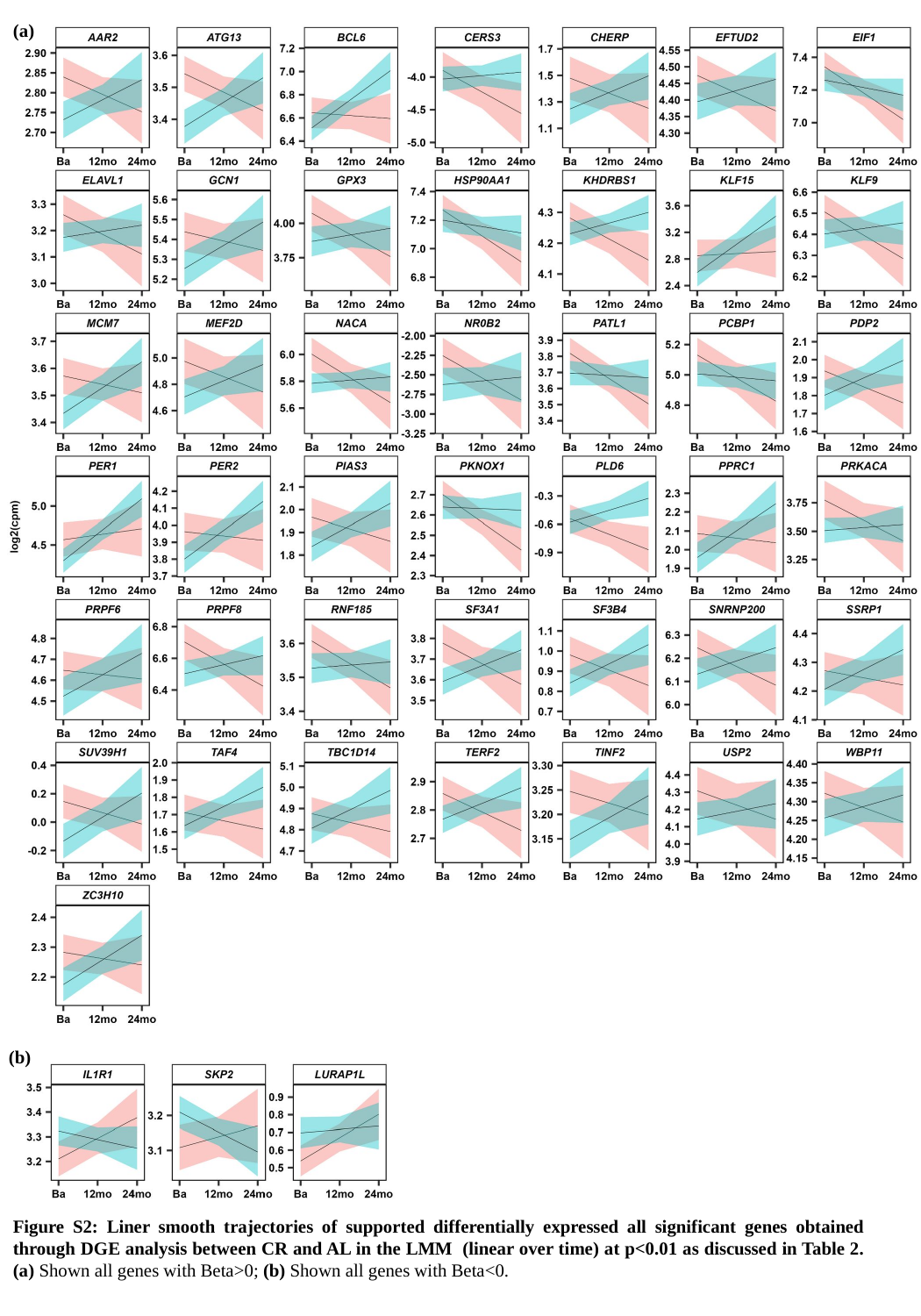

(a)
(b)
Figure S2: Liner smooth trajectories of supported differentially expressed all significant genes obtained through DGE analysis between CR and AL in the LMM (linear over time) at p<0.01 as discussed in Table 2. (a) Shown all genes with Beta>0; (b) Shown all genes with Beta<0.

## Slide 3
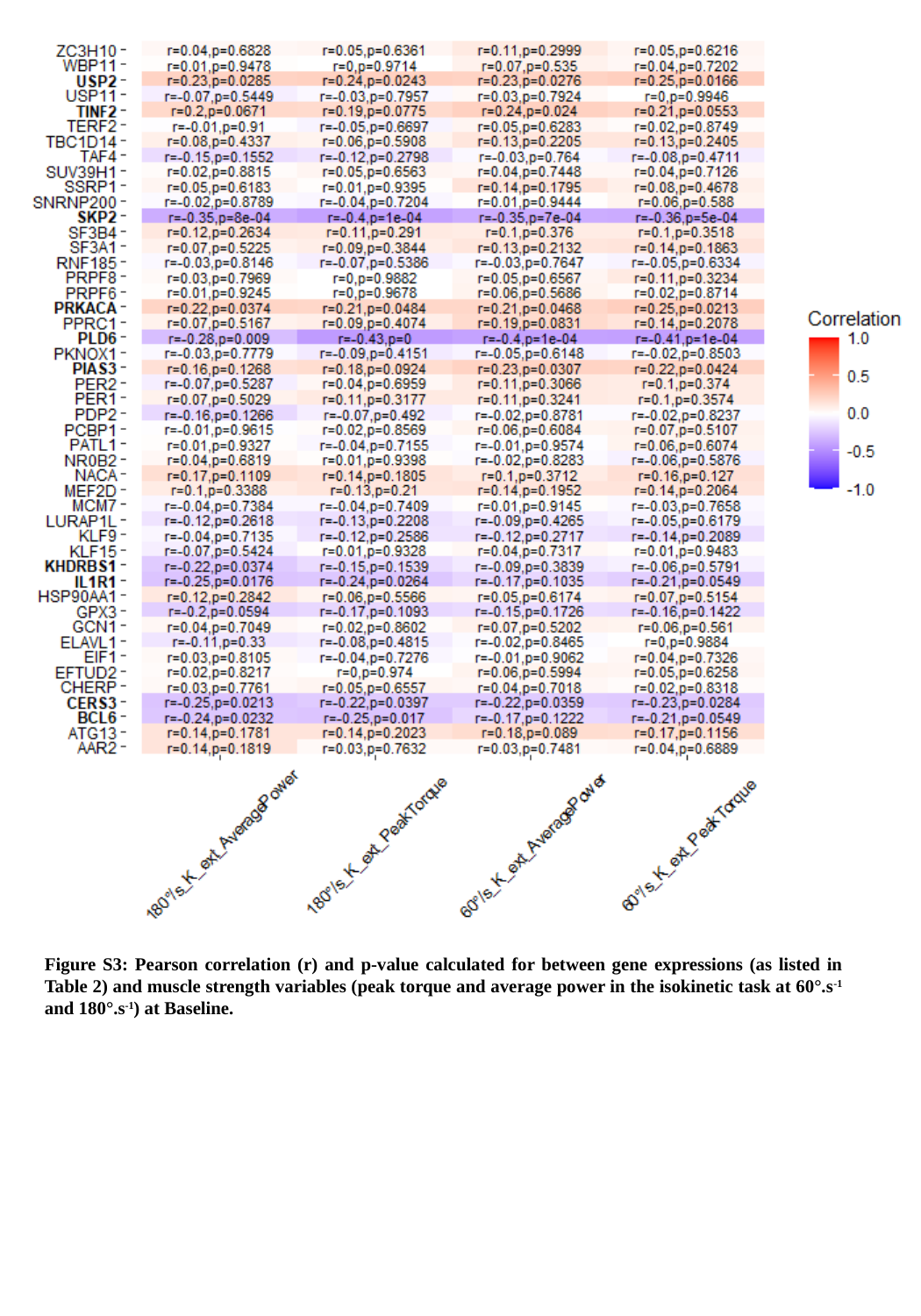

Figure S3: Pearson correlation (r) and p-value calculated for between gene expressions (as listed in Table 2) and muscle strength variables (peak torque and average power in the isokinetic task at 60°.s-1 and 180°.s-1) at Baseline.

## Slide 4
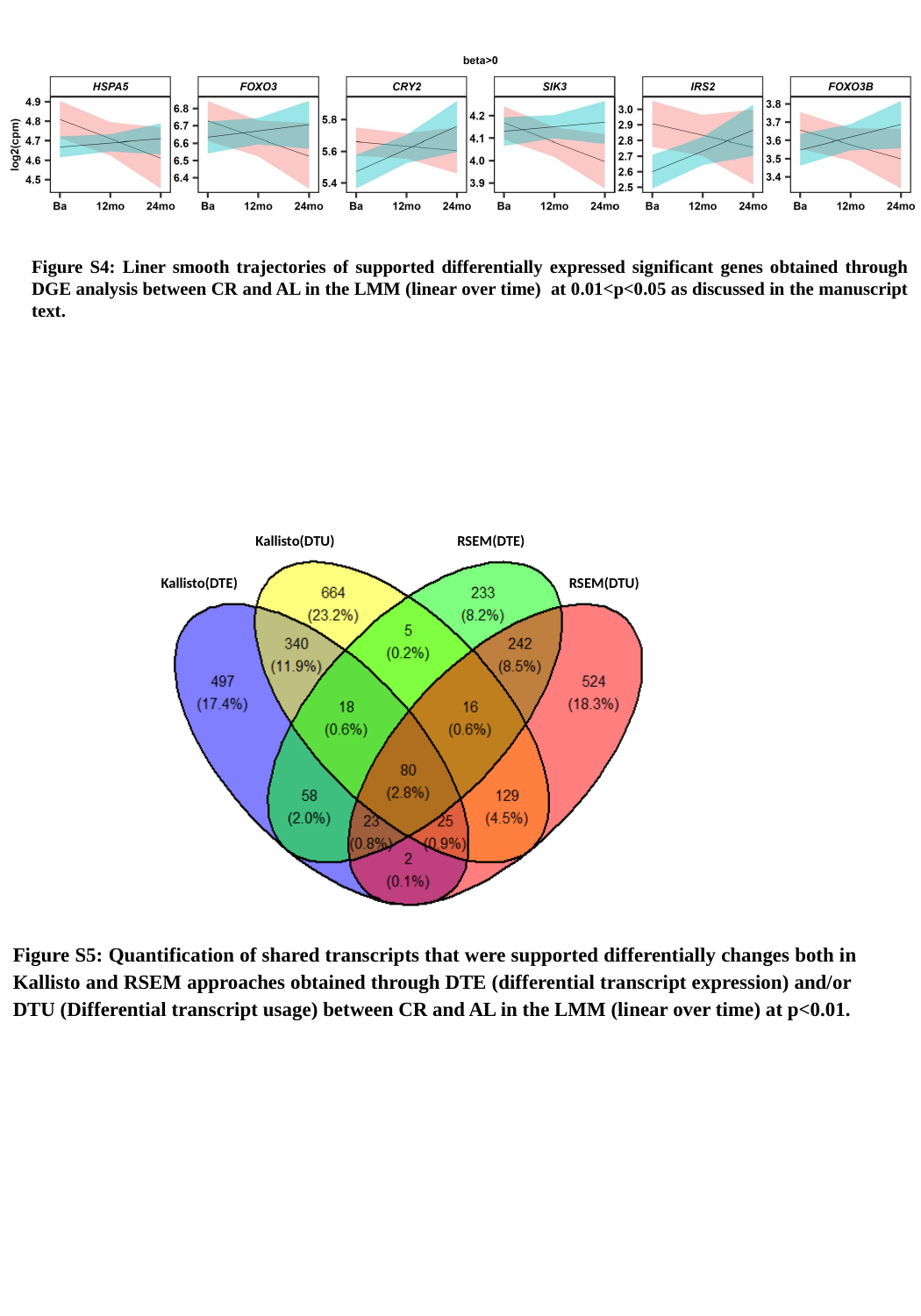

Figure S4: Liner smooth trajectories of supported differentially expressed significant genes obtained through DGE analysis between CR and AL in the LMM (linear over time) at 0.01<p<0.05 as discussed in the manuscript text.
RSEM(DTE)
Kallisto(DTU)
Kallisto(DTE)
RSEM(DTU)
Figure S5: Quantification of shared transcripts that were supported differentially changes both in Kallisto and RSEM approaches obtained through DTE (differential transcript expression) and/or DTU (Differential transcript usage) between CR and AL in the LMM (linear over time) at p<0.01.

## Slide 5
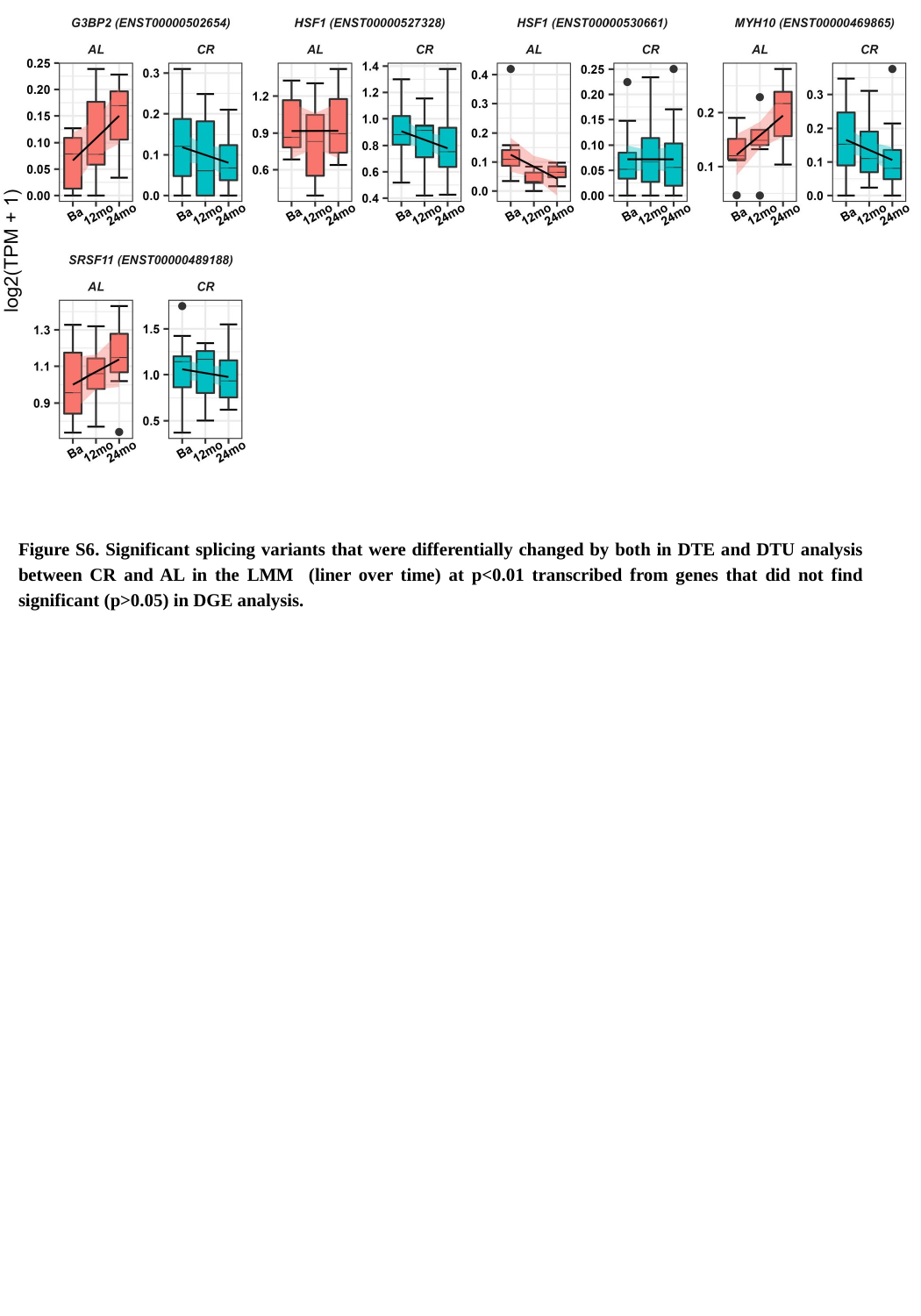

Figure S6. Significant splicing variants that were differentially changed by both in DTE and DTU analysis between CR and AL in the LMM (liner over time) at p<0.01 transcribed from genes that did not find significant (p>0.05) in DGE analysis.
